# MicroRNA-resistant alleles of *HOMEOBOX DOMAIN-2* modify inflorescence branching and increase grain protein content of wheat

**DOI:** 10.1126/sciadv.abn5907

**Published:** 2022-05-11

**Authors:** Laura E. Dixon, Marianna Pasquariello, Roshani Badgami, Kara A. Levin, Gernot Poschet, Pei Qin Ng, Simon Orford, Noam Chayut, Nikolai M. Adamski, Jemima Brinton, James Simmonds, Burkhard Steuernagel, Iain R. Searle, Cristobal Uauy, Scott A. Boden

**Affiliations:** 1 Department of Crop Genetics, John Innes Centre, Norwich Research Park, Norwich NR4 7UH, UK.; 2 Faculty of Biological Sciences, University of Leeds, Leeds LS2 9JT, UK.; 3 School of Agriculture, Food and Wine, Waite Research Institute, University of Adelaide, Glen Osmond, SA 5064, Australia.; 4 Centre of Organismal Studies (COS), University of Heidelberg, Heidelberg 69120, Germany.; 5 School of Biological Sciences, University of Adelaide, Adelaide, SA 5005, Australia.; 6 Germplasm Resources Unit, John Innes Centre, Norwich Research Park, Norwich NR4 7UH, UK.

## Abstract

Plant and inflorescence architecture determine the yield potential of crops. Breeders have harnessed natural diversity for inflorescence architecture to improve yields, and induced genetic variation could provide further gains. Wheat is a vital source of protein and calories; however, little is known about the genes that regulate the development of its inflorescence. Here, we report the identification of semidominant alleles for a class III homeodomain-leucine zipper transcription factor, *HOMEOBOX DOMAIN-2* (*HB-2*), on wheat A and D subgenomes, which generate more flower-bearing spikelets and enhance grain protein content. These alleles increase *HB-2* expression by disrupting a microRNA 165/166 complementary site with conserved roles in plants; higher *HB-2* expression is associated with modified leaf and vascular development and increased amino acid supply to the inflorescence during grain development. These findings enhance our understanding of genes that control wheat inflorescence development and introduce an approach to improve the nutritional quality of grain.

## Teaser

Increased expression of *HB-2* modifies inflorescence architecture and improves grain quality of wheat.

## INTRODUCTION

Bread wheat (*Triticum aestivum*) is a globally important cereal, producing grain that accounts for ~20% of the protein and calories consumed worldwide (*1*). The nutritious grains are produced by florets that develop on lateral branches called spikelets. While the number of spikelets and florets produced by an inflorescence are major determinants of grain yield, very little is known about the genes or biological processes that control their formation (*2*, *3*). This knowledge gap is partially explained by wheat having a complex hexaploid genome that restricts the ability to perform timely genetic analyses of developmental traits (*4*–*6*). However, the recent assembly of reference genome sequences and generation of mutant populations has provided the required resources to identify genes that regulate inflorescence development, opening new opportunities to enhance yield component traits (*5*–*8*).

A typical wheat inflorescence is composed of spikelets arranged in an alternating phyllotaxy on opposite sides of a central rachis and an apical terminal spikelet; each spikelet is subtended by two glumes and commonly produces two to four fertile florets. This arrangement of spikelets is well conserved among wild and cultivated wheat; however, variations do exist that provide an opportunity to identify genes that regulate inflorescence architecture (*9*–*12*). One such variation is the “paired spikelet” that is characterized by the development of two spikelets at an individual rachis node, with a secondary spikelet forming immediately adjacent to and below the regular primary spikelet (*9*, *10*). Paired spikelets are distinct from other variations such as multirow and ramified spikes, which include multiple spikelets or elongated branches at a single rachis node and are promoted by loss-of-function alleles for an APETALA2/ETHYLENE RESPONSIVE FACTOR (AP2/ERF) transcription factor, WHEAT FRIZZY PANICLE (*WFZP*) (*11*, *12*). We have previously interrogated existing genetic variation in cultivated wheat to show that paired spikelet production is a multigenic trait, with at least 18 contributing quantitative trait loci identified using a multiparent advanced generation intercross population (*9*, *10*). We examined one of these loci to show that secondary spikelet formation is facilitated by attenuation of the photoperiod-dependent flowering pathway (*9*). This can be achieved environmentally via growth under noninductive short daylengths or genetically by deletion of the floral activating genes, *Photoperiod-1* (*Ppd-1*) and *FLOWERING LOCUS T1* (*FT1*), which reduces expression of meristem identity genes to promote secondary spikelet formation by delaying the development of the inflorescence and lateral meristems between the double ridge and glume primordium stages (*9*, *10*). Paired spikelet development is also promoted by increased dosage of *TEOSINTE BRANCHED1* (*TB1*), with gene duplication and increased expression of *TB1* inducing paired spikelet formation (*10*). While existing genetic variation has proved useful to identify these roles for *Ppd-1*, *FT1*, and *TB1*, studies in maize, rice, and barley have shown that mutagenesis is a powerful approach to investigate a more complete set of genes that control inflorescence development, which has, so far, been exploited poorly in wheat [e.g., (*13*–*16*)].

Here, we screened for paired spikelet–producing lines within a wheat TILLING (targeting induced local legions in genomes) population to identify genes that regulate wheat inflorescence development (*8*). We identified multiple paired spikelet–producing mutant lines, including two lines that contain mutations in the conserved complementary site for microRNA 165/166 (miR165/166) of a gene encoding a class III homeodomain-leucine zipper (HD-ZIP III) transcription factor. Secondary spikelet formation in these lines is associated with increased expression of the HD-ZIP III transcription factor from both the A and D subgenomes, and the grains produced by these mutants contain more protein than wild-type sibling lines. Our results identify a potential method to improve the grain quality of wheat, and they demonstrate the potential to clone genes controlling the inflorescence development of polyploid wheat using a mutagenesis approach.

## RESULTS

### Identification of a wheat mutant line with modified inflorescence architecture

**Fig. 1.**
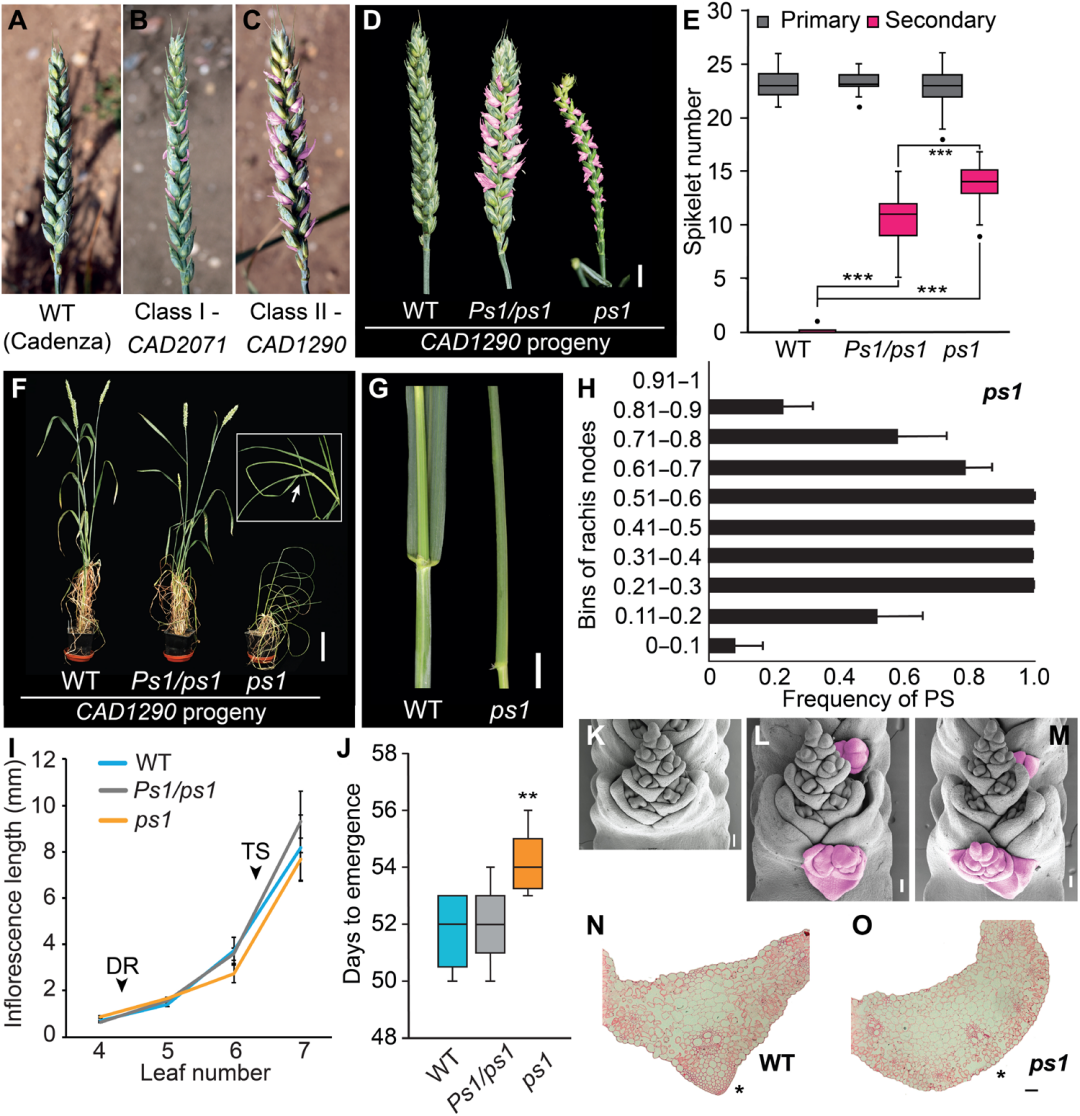
*CAD1290* displays modified spikelet and plant architecture. (**A** to **C**) A screen of the (A) cv. Cadenza wheat mutant population identified (B) class I (e.g., *CAD2071*) and (C) class II (e.g., *CAD1290*) paired spikelet–producing mutant lines. (**D** to **E**) Inflorescences of *ps1* and *Ps1/ps1* (*CAD1290* progeny) produce multiple secondary spikelets (pink), relative to wild-type (WT) siblings. (**F** and **G**) *ps1* plants grown in controlled long daylengths display reduced stature (F) and curled leaves [(F), inset, and (G)], relative to WT. (**H**) The secondary spikelets of *ps1* form predominantly in the central region of the inflorescence: bins 0 to 0.1 and 0.91 to 1 indicate the base and apex of the inflorescence, respectively. (**I**) Inflorescence growth rate and (**J**) flowering time analysis of *Ps1/ps1* and *ps1*, relative to their wild-type sibling line; the timing of double-ridge (DR) and terminal spikelet (TS) stages are indicated by arrows. (**K** to **M**) Scanning electron microscopy analysis shows that secondary spikelets (shown in pink) form on (L) *Ps1/ps1* and (M) *ps1* inflorescences during early developmental stages, but not in WT (K). (**N** and **O**) Safranin-stained cross sections of WT and *ps1* leaves, indicating the abnormal abaxial side of the midrib in *ps1* (highlighted by asterisk). Scale bars, 1 cm (D and G), 10 cm (F), and 100 μm (K to O). (H to J) Data are the average ± SEM of four (I) or eight (H) biological replicates. In the boxplots (E and J), each box is bound by the lower and upper quartiles, the central bar represents the median, and the whiskers indicate the minimum and maximum values of 20 (E) or 8 to 14 (J) biological replicates. **P < 0.01 and ***P < 0.001.

To identify genes that regulate wheat inflorescence development, we explored an ethyl methanesulfonate–induced mutant population (cv. Cadenza) for lines that form paired spikelets (Fig. 1, A to C) (*8*). This TILLING population is suitable for characterizing mutations that promote paired spikelet development because Cadenza was identified previously to form inflorescences without secondary spikelets in multiple field trials (*10*). We identified 256 of 1752 paired spikelet–producing lines that either formed sterile rudimentary secondary spikelets (termed class I mutants; 204 lines; Fig. 1B, fig. S1, and table S1) or multiple secondary spikelets with fertile florets (class II mutants; 52 lines; Fig. 1C, fig. S1, and table S1). Line *CAD1290* was identified as a class II mutant that displayed a particularly strong paired spikelet phenotype; secondary spikelets formed at 47 ± 5.3% of rachis nodes, and many contained fertile florets (Fig. 1, C to E, and fig. S1). The progeny of two *CAD1290* paired spikelet–producing plants was grown under controlled long-day conditions; the inflorescence phenotypes indicated that individuals selected from the field were heterozygous for a dominant or semidominant paired spikelet–promoting allele, as progeny formed either wild-type or paired spikelet–producing inflorescences (Fig. 1, D and E, and tables S2 to S4). Of the progeny that formed secondary spikelets, approximately one-third formed poorly developed inflorescences with multiple infertile florets and curled leaves that failed to completely emerge from the sheath (Fig. 1, F and G, and fig. S1); no plants with wild-type inflorescences displayed the leaf trait.

On the basis of the phenotypes of the *CAD1290* progeny, we hypothesized that paired spikelet development in *CAD1290* is underpinned by a semidominant allele, which confers curled leaves in individuals that are homozygous for the causal mutation. In support of this hypothesis, individuals of the second filial generation derived from two independent *CAD1290* × Cadenza crosses developed wild-type or paired spikelet–producing inflorescences in a ratio of 1:3 (chi-square test, *P* = 0.704 and 0.741), and one-third of the plants with secondary spikelets displayed curled leaves (chi-square test, *P* = 0.931 and 0.864) (table S5). These segregation ratios were confirmed in BC_2_F_2_ and BC_3_F_2_ populations (tables S6 to S8). Homozygous mutant plants with curled leaves produced significantly more paired spikelets than heterozygous individuals that displayed normal leaves (Fig. 1E). On the basis of these results, we conclude that paired spikelet development in *CAD1290* is controlled by a single Mendelian semidominant allele, which promotes leaf curling in homozygous genotypes. We named the mutant *paired spikelet1* (*ps1*) and herein refer to the homozygous and heterozygous mutant genotypes as *ps1* and *Ps1/ps1*, respectively.

Further phenotypic characterization demonstrated that *Ps1/ps1* and *ps1* produced as many primary spikelets per inflorescence as wild-type siblings and that *ps1* inflorescences were significantly shorter than those of wild-type and *Ps1/ps1* (Fig. 1E and fig. S2). Consistent with our previous studies, secondary spikelets were more frequent in the center of the inflorescence for both *Ps1/ps1* and *ps1*, and the fertile secondary spikelets of each genotype contained one or two grain-producing florets, while rudimentary secondary spikelets lacked floral organs (Fig. 1H and fig. S2) (*9*, *10*). Developmental analysis showed that inflorescences of *Ps1/ps1* and *ps1* grew at the same rate as wild type, with no significant delay in the timing of the double-ridge or terminal spikelet stages that define the initiation and completion of spikelet formation (Fig. 1I and fig. S3). This result is consistent with there being no difference in the rachis node number among the three genotypes. The *Ps1/ps1* plants flowered at the same time as wild-type siblings, indicating that secondary spikelet development does not associate with late flowering in these genotypes (Fig. 1J). The *ps1* mutants flowered slightly later than the wild-type and *Ps1/ps1* siblings, most likely because of curling of the flag leaf restricting inflorescence emergence, as there was no delay in flowering when the leaves were unfurled manually (Fig. 1J and fig. S2) Scanning electron microscopy of developing *ps1* and *Ps1/ps1* inflorescences showed that secondary spikelets initiate at the floret primordium stage as a raised cluster of cells that do not contain floral organs and emerged as spikelets with floret primordia at the terminal spikelet stage (Fig. 1, K to M, and fig. S2). The leaf curling of *ps1* was clearly visible at the three-leaf stage and persisted through to flag leaf development (Fig. 1F and fig. S2). Cross sections of the curled leaves showed that the midrib was abnormal, with an absence of lignified sclerenchyma on the abaxial side of the leaf blade (Fig. 1, N and O). The leaf phenotype was initiated during early development, with immature leaves surrounding the developing inflorescence displaying abnormal midribs (fig. S2).

### Identification of a mutant allele for HOMEOBOX DOMAIN-2 that promotes paired spikelet development

**Fig. 2.**
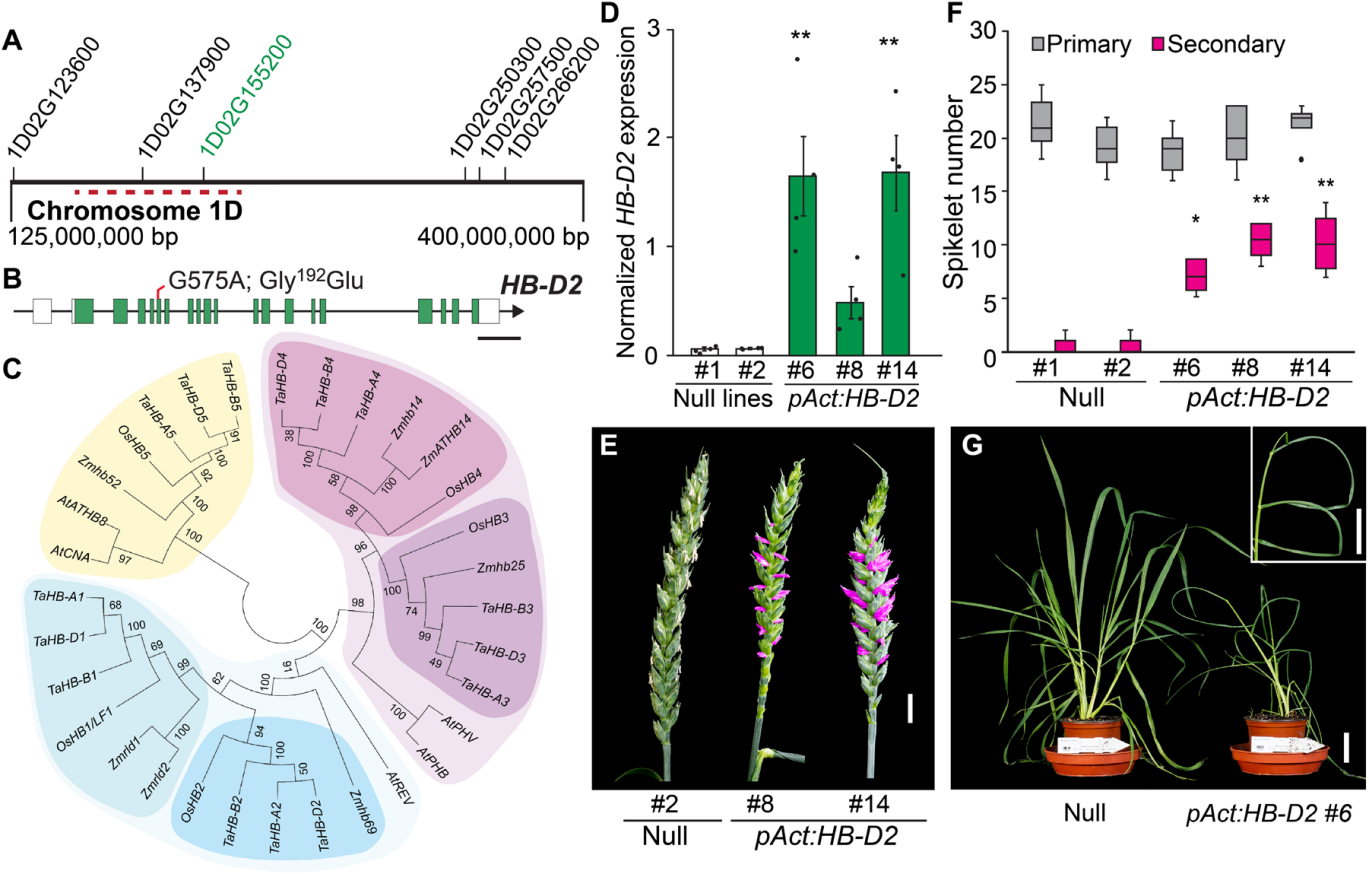
Identification of a mutant allele of *HB-D2* that promotes paired spikelet development and leaf curling. (**A**) Exome capture sequence analysis identified a region on chromosome 1D that associates with paired spikelet development; genes with identified mutations are indicated, including *HB-D2* (highlighted in green; the gene prefix is TraesCS). The region of chromosome 1D investigated further is indicated by dashed red line; the complete list of genes in this region is shown in table S10. (**B**) *HB-D2* gene structure with exons (green), untranslated region (white), introns (black line), and the identified mutation (red line). (**C**) Phylogenetic tree of HD-ZIP III transcription factors in wheat (*Ta*), rice (*Os*), maize (*Zm*), and *Arabidopsis* (*At*). The *HB1/2* clade is shown in blue; *HB3/4* clade is shown in pink, and *HB5* clade is shown in yellow. (**D**) Expression of *HB-D2* in developing inflorescences of *pAct:HB-D2* transgenic lines (T_1_ generation), relative to null control lines. (**E** to **G**) The *pAct:HB-D2* transgenic lines form paired spikelets (secondary spikelets are shown in pink) and curled leaves (see inset) (G). Scale bars, 1 kb (B), 1 cm [(E) and (G), inset], and 5 cm (G). (D) Data are the average ± SEM of four biological replicates. In the boxplot (F), each box is bound by the lower and upper quartiles, the central bar represents the median, and the whiskers indicate the minimum and maximum values of 5 to 10 biological replicates. In (D) and (F), statistical significance is relative to the null control line #1; *P < 0.05 and **P < 0.01.

To identify the mutation responsible for the spikelet and leaf phenotypes, we performed exome capture sequence analysis of DNA bulks composed of either wild-type, *Ps1/ps1*, or *ps1* individuals. The plants for each bulk were selected from second filial generations of two independent lines derived from crossing *Ps1/ps1* to Cadenza (BC_1_F_2_; fig. S4). We filtered for alleles present in the original *CAD1290* parent line, which were absent in the wild-type bulk (i.e., Cadenza-like) and present at a frequency of 0.4 to 0.6 and 0.9 to 1.0 in the *Ps1/ps1* and *ps1* bulks, respectively; fifteen candidate mutations were identified (table S9) (*8*). We then examined six independent segregating populations (564 lines) that had been backcrossed further to Cadenza (BC_2_F_2_) to define a causal region on chromosome 1D (Fig. 2A and table S10). We considered all mutations defined for the *CAD1290* parent line within 79.5 Mb of the identified locus of chromosome 1D and analyzed BC_2_F_3_ and BC_3_F_2–3_ populations (~460 lines) to delimit a 16.5-Mb locus (1D: 216,133,970 to 232,344,836) that included three mutations among 79 genes; failure of mutations to cosegregate with the paired spikelet and leaf phenotypes excluded alleles detected in the original *CAD1290* mutant (table S10) (*8*). From this analysis, we identified a G > A nonsynonymous mutation in *TraesCS1D02G155200* at position 575 base pair (bp) of the coding sequence (Gly^192^Glu), which showed complete cosegregation with *ps1* phenotypes and absolute heterozygosity in *Ps1/ps1* individuals (Fig. 2, A and B, and table S10). *TraesCS1D02G155200* encodes an HD-ZIP III transcription factor, and analysis of RNA sequencing (RNA-seq) data showed that *TraesCS1D02G155200.3* is the splice variant that provides the correct exon-intron structure and encodes a protein homologous to copies on the A and B subgenomes (*TraesCS1A02G157500* and *TraesCS1B02G173900*; Fig. 2B). We named the gene *HOMEOBOX DOMAIN-D2* (*TaHB-D2*) because it is orthologous to *HB-2* from rice and is located on the D genome (Fig. 2C and table S11) (*17*). HB-2 is homologous to REVOLUTA (REV) from *Arabidopsis thaliana* (Fig. 2C) (*18*–*20*). In cereals, the *REV*-*like* genes have been duplicated, and homologs in the sister clade of *HB-2* include *HB-1* of wheat, *rolled leaf1* (*Rld1*) of maize, and *HB1/LATERAL FLORET1* (*OsHB1/LF1*) of rice (Fig. 2C) (*17*, *21*, *22*). Of the other genes encoding HD-ZIP III transcription factors, *HB-3* and *HB-4* are homologous to *PHABULOSA* and *PHAVOLUTA* from *Arabidopsis*, while *HB-5* shares homology with *CORONA* and *ATHB8* (Fig. 2C).

To verify that the identified mutation is causal for the spikelet and leaf phenotypes, we generated transgenic lines (cv. Cadenza) expressing the *ps1* allele of *HB-D2* under control of the *Actin* promoter. The 17 T_0_ transgenic plants that contained the *ps1* allele of *HB-D2* produced paired spikelets, and 8 of 17 formed curled leaves, while null transgenic control lines produced normal leaves and inflorescences without secondary spikelets (fig. S5). We selected three T_1_ transgenic lines that contained one copy of the transgene for further analysis, which each expressed *HB-D2* at higher levels in developing inflorescences than the null control lines (Fig. 2D). Plants of each transgenic line produced inflorescences with multiple paired spikelets (6.25 to 9.90 ± 0.70 to 1.93 per inflorescence) and curled leaves, relative to null control lines that formed normal leaves and inflorescences with only primary spikelets or one to two secondary spikelets per inflorescence 0.40 to 0.50 ± 0.23 to 0.30 per inflorescence; *P* < 0.001; Fig. 2, E to G). There was no significant difference in the number of primary spikelets per inflorescence in the transgenic lines, relative to null control lines, consistent with the phenotypes of *ps1* and *Ps1/ps1* (Fig. 2F). Together, we conclude that the identified mutant allele of *HB-D2* promotes the paired spikelet and leaf curling phenotypes observed in *ps1*.

**Fig. 3.**
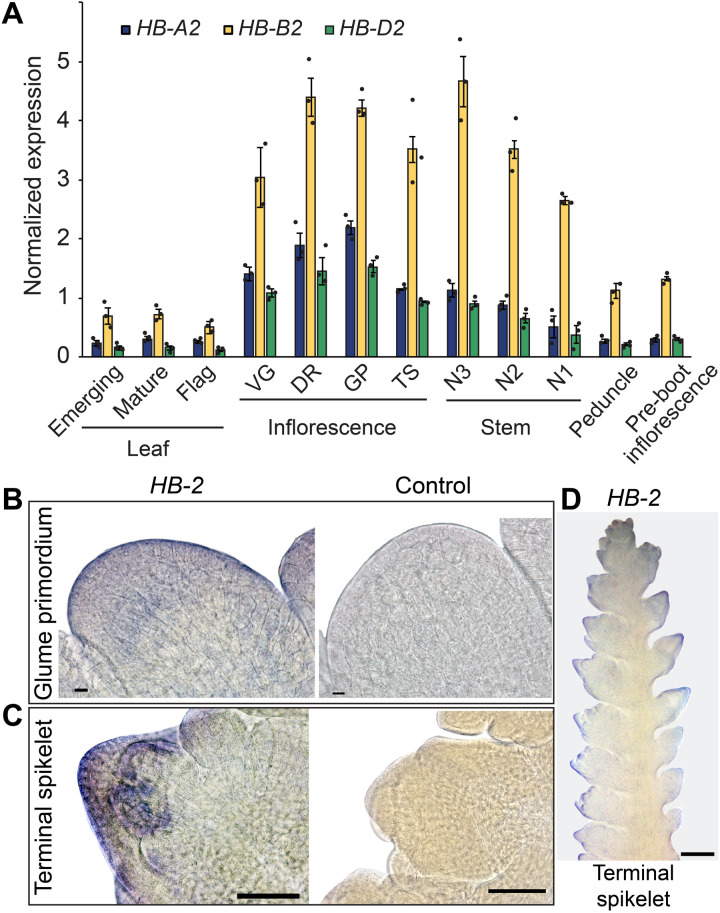
*HB-2* is expressed in the spikelet primordia of developing wheat inflorescences. (**A**) All three homeologs of *HB-2* are expressed strongly in developing inflorescences during early stages when spikelets form and in stem nodes, as well as lowly in leaves and emerging inflorescences. (**B** to **D**) In situ PCR analysis shows that *HB-2* is expressed in the peripheral cell layers of spikelet primordia during the glume primordium (B) and terminal spikelet (C and D) stages. VG, vegetative; DR, double ridge; GP, glume primordium; TS, terminal spikelet; N3, node 3; N2, node 2; N1, node 1. N3 is the basal node, and N1 is the apical node. In (A), data are the average ± SEM of three to four biological replicates. Scale bars, 10 μm (B), 100 μm (C) and 0.5 mm (D).

To investigate a potential role for *HB-D2* during spikelet formation, we analyzed *HB-2* expression during early stages of inflorescence development. Quantitative real-time polymerase chain reaction (PCR) showed that *HB-D2* and its homeologs, *HB-A2* and *HB-B2*, are expressed strongly in developing inflorescences from the vegetative to the terminal spikelet stages, with transcripts peaking at the double-ridge and glume primordium stages that are critical for paired spikelet formation (*9*). *HB-2* transcripts were also detected in stem segments subtending the developing inflorescences, as well as at lower levels in leaves, peduncles, and the emerging mature inflorescence (Fig. 3A). *HB-B2* is the most highly expressed homeolog in each tissue, while *HB-A2* and *HB-D2* transcripts accumulate to similar levels as each other (Fig. 3A). All three homeologs were expressed strongly during the vegetative, double-ridge, glume primordium, and terminal spikelet stages that are critical for spikelet formation, including secondary spikelet development (*9*). In situ PCR analysis resolved the expression of *HB-2* to the peripheral cell layers of the spikelet primordium during the glume primordium and terminal spikelet stages, which are developmental stages critical for primary and secondary spikelet formation (Fig. 3, B to D) (*9*). The spatiotemporal expression pattern is consistent with *HB-2* performing a role during spikelet formation, including stages of inflorescence development critical for paired spikelet production (*9*).

### Independent microRNA-resistant alleles of *HB-2* promote paired spikelet development

**Fig. 4.**
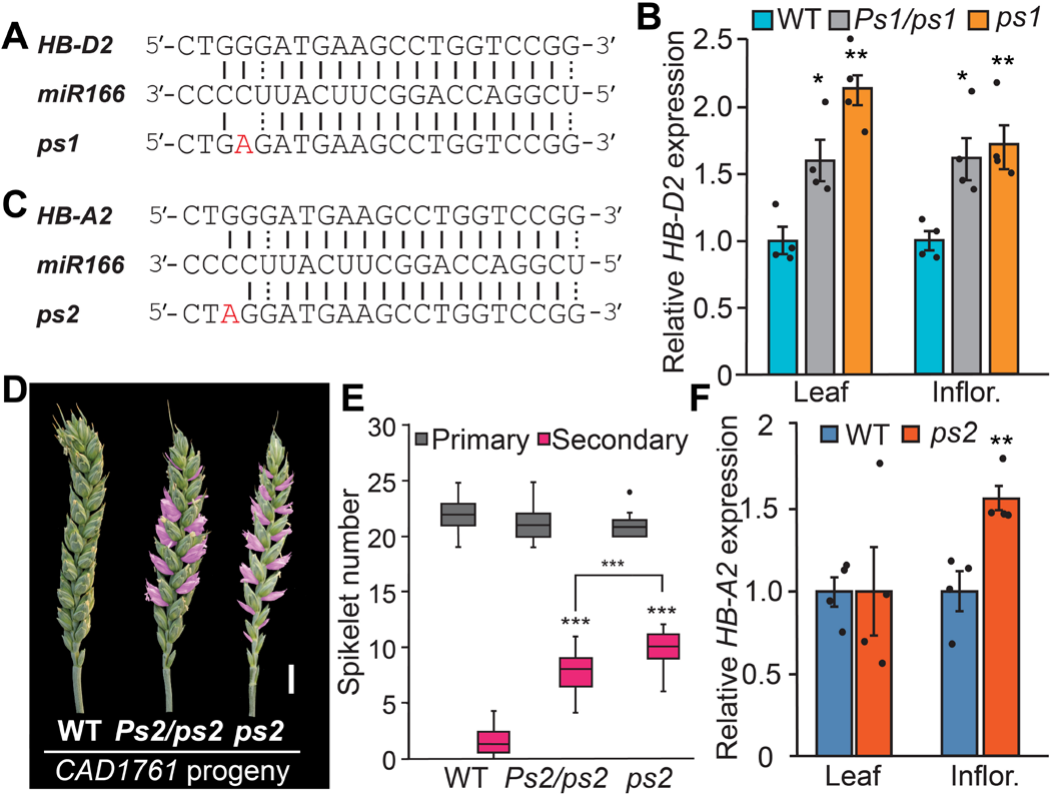
Mutations in the microRNA complementary site of *HB-2* promote paired spikelet development. (**A**) The identified G > A mutation (red text) of *ps1* is in the miR165/166 complementary site of *HB-D2*. (**B**) *HB-D2* is expressed higher in leaves and developing inflorescences (Inflor.) of *Ps1/ps1* and *ps1* lines, relative to wild-type siblings. (**C**) An independent class II paired spikelet–producing mutant (*CAD1761*), named *ps2*, contains a G > A mutation (red text) in the miR165/166 complementary site of *HB-A2*. (**D** and **E**) Heterozygous and homozygous *ps2* mutants form paired spikelets (secondary spikelets are shown in pink) and (**F**) express *HB-A2* significantly higher in developing inflorescences but not in leaves. (B and F) Data are the average ± SEM of four biological replicates. In the boxplot (E), each box is bound by the lower and upper quartiles, the central bar represents the median, and the whiskers indicate the minimum and maximum values of eight biological replicates. Scale bar, 1 cm (D). *P < 0.05, **P < 0.01, and ***P < 0.001.

The curled leaves of *ps1* resemble the rolled leaves produced by the semidominant *Rld1-Original* (*Rld1-O*), *OSHB1m/lateral floret1*, and *rev-10d*/*avb1* mutants of maize, rice, and *Arabidopsis*, respectively (*17*, *18*, *20*, *21*, *23*). *Rld1*, *HB1*, and *REV* transcripts accumulate in the respective mutants because mRNA cleavage is disrupted by mutations in a miR165/166 complementary site (*20*–*22*). Given that *ps1* forms similar curled leaves, we asked whether the identified mutation in *HB-D2* was positioned in the complementary site for *miR165*/*166*. The identified mutation was located at the miR165/166 complementary site of *HB-D2*, changing the same nucleotide as that altered in the corresponding site of *Rld1* in the maize *Rld1-O* mutant (Fig. 4A) (*21*). Quantitative transcript analysis showed that *HB-D2* was expressed significantly higher in leaves and developing inflorescences of *Ps1/ps1* and *ps1*, relative to wild-type siblings, consistent with the identified mutation disrupting the miR165/166-guided cleavage of *HB-D2* transcripts (Fig. 4B). In situ PCR analysis of developing *ps1* inflorescences showed that *HB-D2* was expressed in the same region of the spikelet primordia as determined for wild type, indicating that the higher transcript levels are not due to ectopic expression within developing spikelets (fig. S6)*. miR166* was detected in developing inflorescences at comparable levels in *ps1* and wild type, indicating that *miR166* regulates *HB-D2* expression during spikelet formation and that reduced *miR166* levels are not responsible for the higher level of *HB-D2* transcripts in *ps1* (fig. S6).

To further investigate the effect of increased *HB-2* expression on secondary spikelet formation, we asked whether any independent class II paired spikelet mutants from our screen of the TILLING population contained mutations in the miR165/166 complementary site of *HB-2* (*8*). Line *CAD1761*, which displayed a strong paired spikelet phenotype, carried a G > A nonsynonymous mutation in the miR165/166 complementary site of *HB-A2* (580 bp; G > A) that neighbors the nucleotide altered in *HB-D2* of *ps1* (Fig. 4, C to E). Exome capture sequence analysis showed that this mutation is associated with paired spikelet formation, with the mutant allele detected in the bulked DNA of plants with paired spikelets (144 of 169 reads) and not in the wild-type DNA bulk (0 of 188 reads). Generation and analysis of two independent backcrossed segregating families (BC_2_F_2_) confirmed that the mutant *HB-A2* allele is associated with secondary spikelet development and is inherited as a single semidominant Mendelian locus (chi-square test, *P* = 0.71 and 0.64) (Fig. 4, D and E, and table S12). Both heterozygous and homozygous mutants, but not wild-type siblings, formed paired spikelets; the homozygous and heterozygous genotypes were named *ps2* and *Ps2/ps2*, respectively (Fig. 4, D and E). *HB-A2* transcripts were significantly higher in developing inflorescences of *ps2*, relative to wild type (Fig. 4F), providing independent genetic evidence that mutation of the miR165/166 complementary site of *HB-2* promotes paired spikelet development by increasing *HB-2* expression. As with *ps1* mutants, the secondary spikelets of *ps2* formed predominantly within the central region of the inflorescence and emerged during the floret primordium and terminal spikelet stages (fig. S7). Similarly, *ps2* formed as many primary spikelets as wild-type siblings, and there were no significant differences in flowering time or inflorescence growth rates (Fig. 4E and fig. S7). The *ps2* plants did not, however, form curled leaves nor was *HB-A2* expressed higher in *ps2* leaves, suggesting that *HB-2* homeologs have slightly different functions (Fig. 4F). Together with the analysis of *ps1* and the transgenic lines, these results show that paired spikelet formation is promoted by increased expression of *HB-2* homeologs on the A and D subgenomes, which can be facilitated by modifying bases in the miR165/166 complementary site.

### The photoperiod-dependent flowering pathway enhances paired spikelet production in *ps1* and *ps2*

**Fig. 5.**
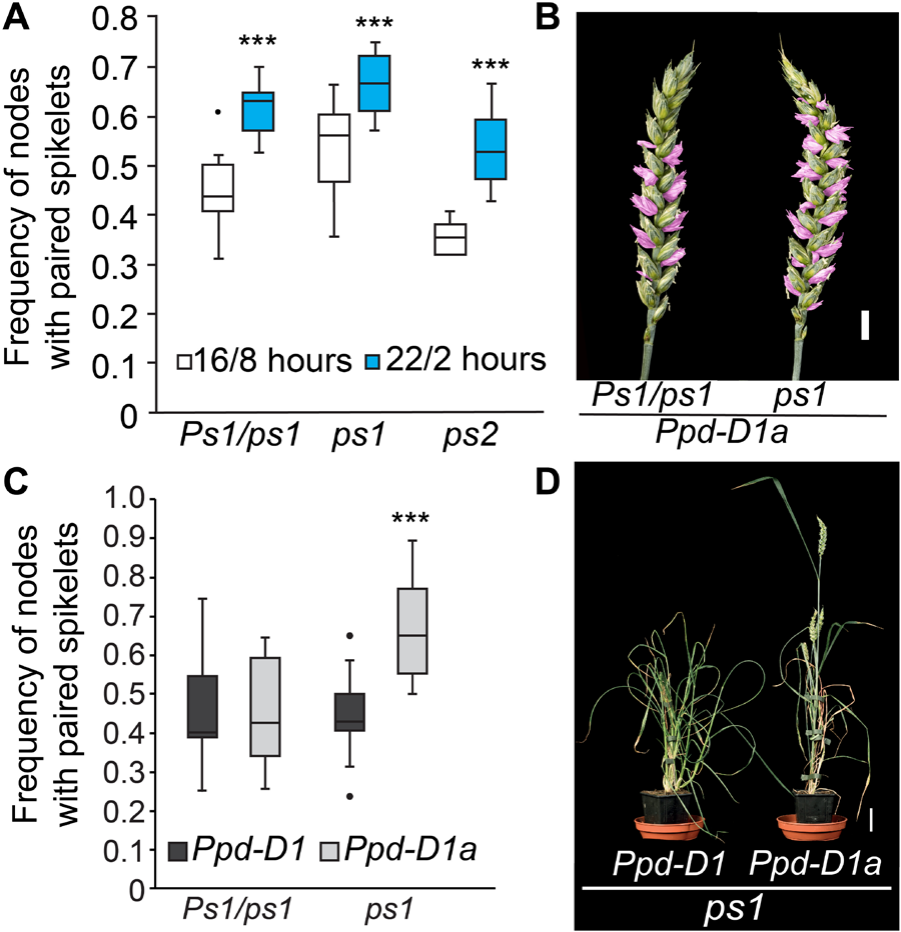
Environmental and genetic analysis of the interaction between *HB-2* and the photoperiod-dependent flowering pathway. (**A**) The *Ps1/ps1*, *ps1*, and *ps2* mutants form significantly more paired spikelets under extra-long daylengths (22/2 hours) relative to standard long-day conditions (16/8 hours). (**B** to **D**) Phenotypic analysis of (B and C) inflorescence and (D) plant architecture traits of the *Ps1/ps1* and *ps1* lines that express the photoperiod-insensitive *Ppd-D1a* allele, relative to photoperiod sensitive sibling lines (*Ppd-D1*). In the boxplots (A and C), each box is bound by the lower and upper quartiles, the central bar represents the median, and the whiskers indicate the minimum and maximum values of 12 to 24 biological replicates. Scale bars, 1 cm (B) and 5 cm (D). ***P < 0.001.

In wheat, photoperiods and genotypes that induce a strong flowering signal typically reduce the number of spikelets that form per inflorescence, and these conditions also inhibit secondary spikelet development (*9*, *10*, *24*–*26*). To determine whether the photoperiod-dependent flowering pathway influences secondary spikelet development in *ps1*, *Ps1/ps1*, and *ps2*, we grew these genotypes under extended daylengths that induce strong expression of *FT1* (*10*). Unexpectedly, growth under extralong photoperiods (22/2 hours) significantly enhanced secondary spikelet production in *Ps1/ps1*, *ps1*, and *ps2* mutants, relative to 16-hour photoperiods, demonstrating that paired spikelet production in these genotypes is not diminished by conditions that induce a strong floral promoting signal (Fig. 5A). We then investigated the influence of the photoperiod-dependent flowering pathway by generating *ps1* genotypes that contain the photoperiod-insensitive *Ppd-D1a* allele, which induces strong *FT1* expression and suppressed secondary spikelet formation in other genotypes (*9*). As expected, both heterozygous and homozygous mutant lines with the photoperiod-insensitive *Ppd-D1a* allele produced fewer primary spikelets than sibling lines that contained the photoperiod-sensitive allele (fig. S8). However, expression of the *Ppd-D1a* allele in *ps1* enhanced paired spikelet development, relative to photoperiod-sensitive sibling lines, and it improved the performance of *ps1* by advancing leaf and inflorescence emergence (Fig. 5, B to D). In the *Ps1/ps1* genotype, the photoperiod-insensitive *Ppd-D1a* had no significant effect on secondary spikelet formation (Fig. 5C). In support of these results, photoperiod-insensitive elite cultivars (e.g., cv. Mace, Rockstar, and Sheriff) produced paired spikelets following the introduction of the *ps1* and *ps2* alleles of *HB-2* (fig. S9). Together, these results show that the environmental conditions that induce a strong floral-promoting signal enhance paired spikelet production in lines that express microRNA-resistant alleles of *HB-2*, and photoperiod-insensitive alleles of *Ppd-1* do not suppress secondary spikelet formation in *ps1* and *ps2* as they do in other genotypes.

### The transcriptome of a developing inflorescence is modified in *ps1* and *ps2* mutants

**Fig. 6.**
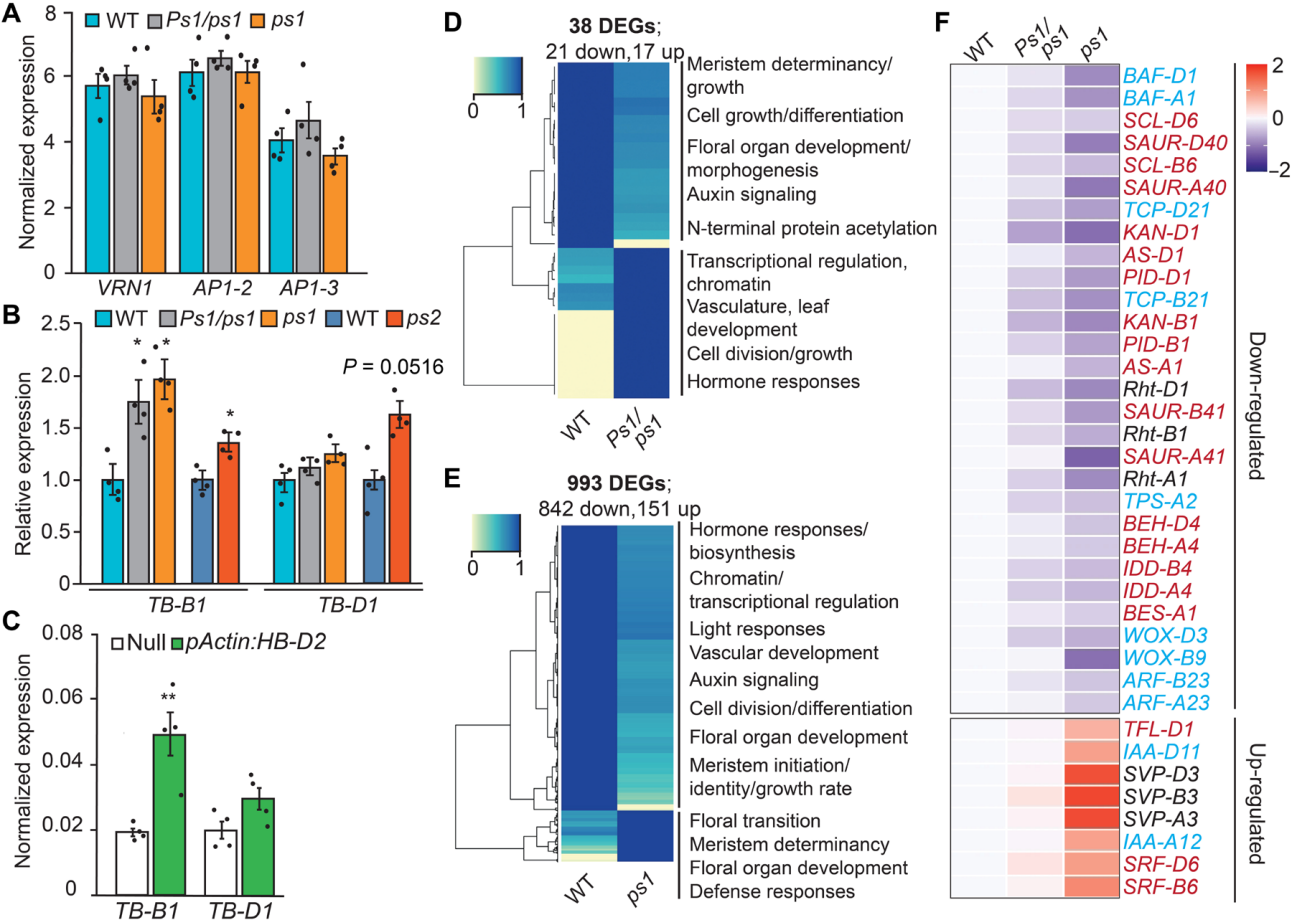
Analysis of gene expression during early inflorescence development. (**A**) Spikelet meristem identity genes identified to be differentially expressed in other paired spikelet–producing genotypes are not significantly different in *Ps1/ps1* and *ps1* relative to wild-type siblings. (**B** and **C**) *TB-B1* is more highly expressed in *Ps1/ps1*, *ps1*, and *ps2* mutants relative to their respective wild-type siblings and in *pAct:HB-D2* transgenic lines compared to null control lines. (**D** and **E**) Heatmaps of DEGs identified in developing inflorescences of (D) *Ps1/ps1* and (E) *ps1* relative to wild-type siblings. Identified GO terms are indicated at the left of the heatmap. For down-regulated DEGs, TPM values are normalized to wild type that is set at 1, and for up-regulated DEGs, the *Ps1/ps1* and *ps1* TPM value is set at 1. (**F**) Heatmap of DEGs in developing inflorescences of *ps1* that have been identified in wheat (black text), maize (blue text), or *Arabidopsis* (red text) to have roles during inflorescence/floral or leaf development. Data of *Ps1/ps1* and *ps1* are normalized TPM values of three biological replicates shown as a log_2_ fold changes relative to WT. *P < 0.05 and **P < 0.01.

Our previous work showed that paired spikelet formation is facilitated by increased dosage of *TB1* and reduced expression of spikelet meristem identity genes during early inflorescence development (*9*, *10*). To determine whether the expression of spikelet meristem identity genes is modified similarly in *Ps1/ps1*, *ps1*, and *ps2*, relative to wild-type sibling lines, we quantified transcripts of *VERNALIZATION1* (*VRN1*), *APETALA1-2* (*AP1-2*; previously named *AGL10*), *AP1-3* (previously named *AGL29*), and *SEPALLATA1-3* (*SEP1-3*; previously named *AGLG1*) during early inflorescence development (*9*, *10*). Transcripts of *VRN1*, *AP1-2*, *AP1-3*, and *SEP1-3* were not significantly reduced in *Ps1/ps1*, *ps1*, or *ps2*, relative to wild-type siblings; these results indicate that secondary spikelets form in these genotypes via a mechanism different from that identified in *ppd-D1* and *ft-B1* mutants (Fig. 6A and fig. S10). We then quantified transcripts of *TB-B1* and *TB-D1*, as increased expression of these two homeologs facilitates paired spikelet development (*10*). Transcripts of *TB-B1* were significantly higher in *ps1*, *Ps1/ps1*, and *ps2*, relative to their respective wild-type sibling lines, while those of the less abundant *TB-D1* were slightly, but not significantly, increased in the mutant lines (Fig. 6B). In support of these results, *TB-B1* expression was significantly higher in inflorescences of *HB-D2* transgenic plants, relative to the null control line, while *TB-D1* transcripts were slightly, but not significantly, greater in the transgenic line (Fig. 6C). Together with our previous analysis of TB1-dependent regulation of paired spikelet development, these results indicate that increased expression of *TB-B1* contributes to secondary spikelet formation in the *ps1* and *ps2* mutant lines.

To further investigate biological processes influenced by higher expression of *HB-D2*, we used RNA-seq analysis to compare the transcriptome of developing inflorescences from wild-type plants to those of *Ps1/ps1* and *ps1*. We detected a similar number of expressed genes for each of the three genotypes based on expression of >0.5 transcripts per million [TPM; wild type expressed 47,628 high-confidence (HC) genes and 22,979 low-confidence (LC) genes; *Ps1/ps1* expressed 47,886 HC and 24,654 LC; *ps1* expressed 47,885 HC and 23,383 LC]. Differential gene expression analysis detected 151 up-regulated (133 HC and 18 LC) and 842 down-regulated (743 HC and 99 LC) genes in *ps1*, relative to wild type (993 total), while 17 (16 HC and 1 LC) and 21 (20 HC and 1 LC) transcripts were significantly up- and down-regulated in *Ps1/ps1* (total, 38) (Fig. 6, D and E, and data file S1). While fewer differentially expressed genes (DEGs) were detected in *Ps1/ps1*, many DEGs of *ps1* showed a conserved response in *Ps1/ps1*, relative to wild-type siblings (Fig. 6F and data file S1). The RNA-seq analysis confirmed that *HB-D2* transcripts were more abundant in *Ps1/ps1* and *ps1*, relative to wild type, and showed that *HB-2* is the most highly expressed HD-ZIP III transcription factor during early inflorescence development (fig. S10). The gene ontology (GO) term analysis of DEGs in *ps1* showed that down-regulated genes were enriched for biological processes associated with spikelet formation, including meristem initiation and growth, floral organ development, auxin signaling, and cell differentiation (Fig. 6, D and E, and data file S1). Similarly, up-regulated genes were enriched for GO terms related to the floral transition and meristem determinacy (Fig. 6, D and E). The identified DEGs include homologs of genes from *Arabidopsis*, rice, or maize that perform roles during inflorescence development, vasculature formation, and floral transition (Fig. 6F). For example, up-regulated genes include homologs of *STRUBBELIG-RECEPTOR FAMILY6/7* (*SRF6/7*) (*27*, *28*) and *SHORT VEGETATIVE PHASE-3*, which is a member of a gene family that regulates spikelet architecture (e.g., glume length) and fertility in wheat and is up-regulated in rice mutants that form highly branched panicles (*16*, *29*–*34*) (Fig. 6F). We did not detect increased expression of *WKNOX* homeologs, which was supported by quantitative reverse transcription PCR (qRT-PCR) analysis; *WKNOX* is the wheat ortholog of *OSH1* (*ORYZA SATIVA HOMEOBOX 1*) that was up-regulated in the rice *lf1* mutant (fig. S10) (*22*, *35*). Homologs of down-regulated genes include those that influence inflorescence architecture (e.g., *BARREN STALK FASTIGIATE1*, *GNAT-like HISTONE ACETYLTRANSFERASE1*, and *PINOID/BARREN INFLORESCENCE2*) (*36*–*39*); auxin and brassinosteroid signaling/responses (e.g., *AUXIN TRANSPORTER-LIKE PROTEIN 2*, *AUXIN RESPONSE FACTOR3*, *BRI1 EMS SUPPRESSOR*, and *BES HOMOLOGUE4*) (*40*–*42*); floret, leaf, and vasculature development (e.g., *INDETERMINATE DOMAIN4* and *KANADI1*) (*43*, *44*); and cell growth (e.g., Reduced Height-1; Fig. 6F) (*45*). Together, these results indicate that paired spikelet development in *Ps1/ps1* and *ps1* is associated with altered expression of genes that influence the determinacy, differentiation, and growth of axillary spikelet meristems during early inflorescence development.

### Increased *HB-2* expression facilitates higher grain protein content

**Fig. 7.**
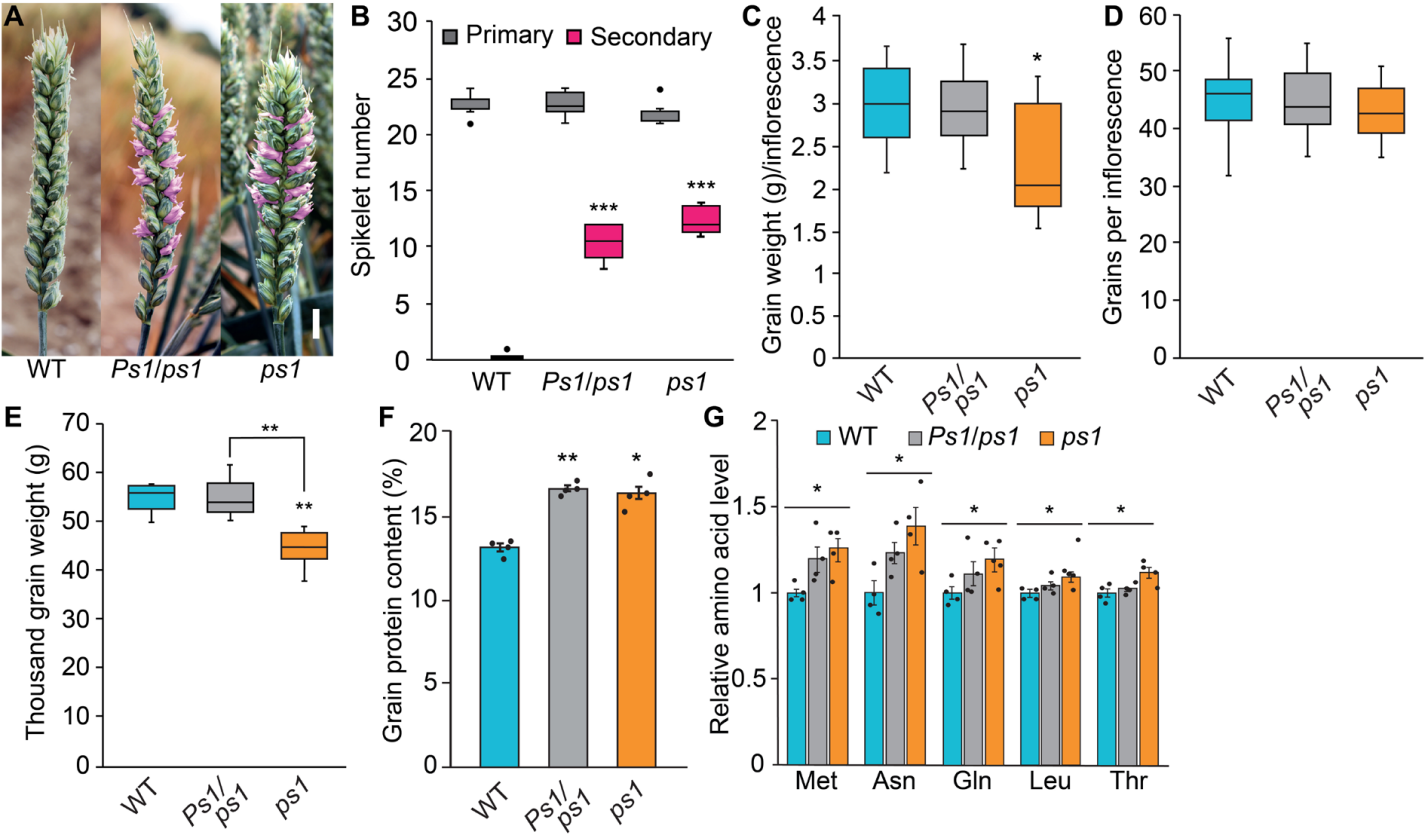
Analysis of yield and grain quality traits of field-grown *Ps1/ps1* and *ps1* plants. (**A** and **B**) Field-grown *Ps1/ps1* and *ps1* plants form multiple secondary spikelets (pink) relative to wild-type siblings. (**C** to **E**) The weight and number of grain per inflorescence, and thousand grain weight, of *Ps1/ps1* and *ps1* plants relative to wild-type siblings. (**F** and **G**) The grain produced by *Ps1/ps1* and *ps1* have higher levels of protein (F) and free amino acids (mg/g of flour) than those produced by wild-type siblings. In (F) and (G), data are the average ± SEM of four biological replicates. In the boxplots, each box is bound by the lower and upper quartiles, the central bar represents the median, and the whiskers indicate the minimum and maximum values of six to eight biological replicates. Scale bar, 1 cm (A). *P < 0.05, **P < 0.01, and ***P < 0.001.

To investigate the effect of the modified inflorescence architecture on yield-related traits and the relevance of the *Ps1/ps1* and *ps1* lines to breeding, we examined the yield and quality of grain produced by plants grown under field conditions. Field-grown *Ps1/ps1* and *ps1* produced inflorescences with multiple paired spikelets, and there was no significant difference in primary spikelet number among the three genotypes (Fig. 7, A and B). Unexpectedly, *ps1* plants performed better in the field than the glasshouse, growing to a similar height and producing as many tillers as wild-type siblings; the improved performance is likely due to a delayed rate of leaf appearance and inflorescence development in the field, relative to plants grown under warmer glasshouse conditions (fig. S11) (*25*). The *Ps1/ps1* and *ps1* lines flowered at the same time as wild-type siblings, and there was no significant difference in the timing of senescence (fig. S11). In terms of yield-related traits, the heterozygous *Ps1/ps1* lines produced the same weight and number of grains per inflorescence as wild-type siblings, and there was no significant difference in the thousand grain weight, grain size, yield per plant, or harvest index (Fig. 7, C to E, and fig. S11). In *ps1*, the thousand grain weight, grain size, and weight per inflorescence was slightly lower than wild type, but the grain number per inflorescence and yield per plant were comparable to wild-type siblings (Fig. 7, C to E, and fig. S11). Regarding grain quality, we focused on grain protein content (GPC) as it is a major determinant of wheat’s nutritional value (*1*). The grain of *Ps1/ps1* and *ps1* contained ~25% more protein than wild type, as well as higher levels of free amino acids, including the essential methionine, leucine, and threonine (Fig. 7, F and G). The grain of *Ps1/ps1* and *ps2* also contained more protein (21 and 21%, respectively) when grown under glasshouse conditions, relative to their respective wild-type siblings; protein content was 130% higher for glasshouse-grown *ps1* plants, but this is likely to be due to the low-yielding inflorescences of *ps1* when grown under these conditions (fig. S12). Together, these results indicate that increased expression of *HB-2* facilitates the production of grain with higher protein content without significantly affecting grain yield.

**Fig. 8.**
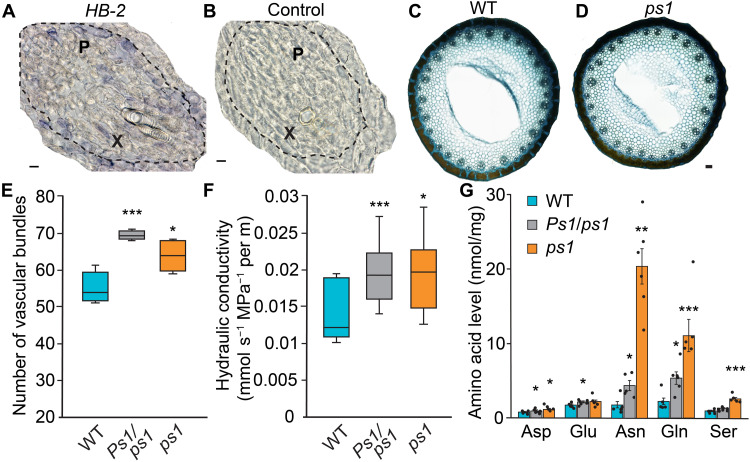
Analysis of plant vasculature, hydraulic conductivity, and rachis amino acid content. (**A** and **B**) In situ PCR analysis shows that *HB-2* is expressed in vascular bundles of the stem [bordered by dashed line, with regions of xylem (X) and phloem (P) indicated] and in cells surrounding the vasculature. (B) A negative control of the in situ PCR analysis. (**C** and **D**) Toluidine blue–stained cross sections of peduncles from (C) *ps1* mutants relative to its wild-type sibling (D). (**E**) Stems of *Ps1/ps1* and *ps1* plants contain more vascular bundles than wild-type siblings. (**F**) Analysis of hydraulic conductivity in the peduncle and mature inflorescence of *Ps1/ps1* and *ps1* relative to its wild-type sibling. (**G**) Levels of abundant amino acids are higher in rachises of *Ps1/ps1* and *ps1* relative to wild-type siblings (WT). In the boxplots (E and F), the box is bound by the lower and upper quartiles, the central bar represents the median, and whiskers indicate the minimum and maximum values of (E) five to eight and (F) seven biological replicates. (G) Data are the average ± SEM of six biological replicates. Scale bars, 10 μm (A) and 100 μm (B and D).

To investigate further how *Ps1/ps1*, *ps1*, and *ps2* plants produce grain with more protein, we asked whether *HB-2* influences vascular development in stems similarly to *REV* in *Arabidopsis* (*18*, *20*, *46*) such that more assimilates can be delivered to the inflorescence and developing grain. In *Arabidopsis*, the vascular bundles of the semidominant *rev-10d*/*avb1* mutants localize more centrally in the stem and display a radialized pattern, as opposed to the wild-type collateral structure (*18*, *20*). In situ PCR analysis supported *HB-2* performing a role in wheat vascular bundle formation, as *HB-2* transcripts were detected in cells surrounding the xylem and phloem of stems and in the immediately adjacent cells (Fig. 8, A and B, and fig. S13). Analysis of peduncle cross sections showed that the vasculature in each genotype were arranged in two rings surrounding a central pith, and the stems of *Ps1/ps1*, *ps1*, and *ps2* contained significantly more vascular bundles than their respective wild-type sibling lines (Fig. 8, C to E, and fig. S13). On the basis of these results, we hypothesized that the extra vascular bundles in *ps1* and *ps2* may facilitate greater distribution of assimilates to the inflorescence and developing grain. To test this hypothesis, we analyzed the hydraulic conductance of each genotype as grain formed in mature florets. The hydraulic conductivity of the peduncle and inflorescence from *Ps1/ps1*, *ps1*, and *ps2* was greater than that of their respective wild-type siblings, indicating that lines with increased expression of *HB-2* can distribute more assimilates to the developing grain (Fig. 8F and fig. S13). This conclusion is supported by the metabolic analysis of *Ps1/ps1* and *ps1* rachises, which contained significantly higher amounts of asparagine, aspartic acid, glutamine, and serine relative to the wild-type sibling; these amino acids are the most abundant in wheat vasculature (Fig. 8G) (*47*). Levels of scarcer amino acids, including histidine, arginine, valine, isoleucine, leucine, and lysine were also significantly higher in rachises of *Ps1/ps1* and *ps1* relative to wild type (fig. S13). Together, these results suggest that increased expression of *HB-2* facilitates the production of grain with more protein by modifying vascular development, which increases the supply of assimilates to the inflorescence and developing grain.

## DISCUSSION

Inflorescence architecture is a major determinant of grain production, with spikelet number and floret fertility contributing significantly to yield (*2*, *48*, *49*). Here, we sought to identify genes that control spikelet development by exploring a TILLING population for paired spikelet–producing lines (*8*). We identified independent lines that contain mutations in the miR165/166 complementary sites of *HB-2* on the A and D subgenomes, which promote paired spikelet formation by increasing *HB-2* expression in the developing inflorescence. The increased expression is consistent with mutations in microRNA complementary sites disrupting the cleavage of the target transcript and with the identified alleles being semidominant for secondary spikelet formation (*17*, *18*, *20*–*22*, *50*). Discovery of these alleles fits with the high likelihood of identifying dominant or semidominant mutations in wheat given the redundancy of its hexaploid genome (*51*). They join *Q* of the domesticated wheat as microRNA-resistant alleles that modify inflorescence architecture by increasing levels of the target transcript (*52*, *53*). While identification of these alleles is consistent with expectations for a polyploid species, our previous analysis of a *ft-B1* mutant shows that loss-of-function mutations within a single homeolog can facilitate paired spikelet development; additional examples are likely to involve genes for which there is biased expression of a homeolog from a particular subgenome (*9*, *54*). Further analysis of the paired spikelet–producing mutants identified here will help identify genes that regulate inflorescence architecture and determine the potential to use both dominant and recessive alleles to investigate wheat development.

The identification of microRNA-resistant *HB-2* alleles provides new understanding of the developmental processes that influence spikelet architecture in wheat. Our previous analyses of *ppd-D1*, *ft-B1*, and *highly branched* lines showed that genotypes and environments that promote strong floral-promoting signals repress secondary spikelet formation, while those that induce weak expression of *FT1* and spikelet meristem identity genes facilitate paired spikelet development (*9*, *10*). Contrary to these studies, the secondary spikelet formation of *ps1* and *ps2* was enhanced by extralong daylengths, and photoperiod-insensitive genotypes that promote *FT1-*dependent early flowering did not suppress paired spikelet development in *ps1*, *Ps1/ps1*, or *ps2*. Moreover, expression of genes that promote spikelet meristem identity was not reduced in developing inflorescences of *ps1* and *ps2*. Together with the analysis of inflorescence development, these results indicate that paired spikelet formation in *ps1* and *ps2* is not caused by a delay in spikelet meristem maturation, as described for other genotypes (*9*, *10*). Paired spikelet production in *ps1* and *ps2* is, however, associated with higher *TB1* expression, which indicates that increased dosage of *TB1* may facilitate secondary spikelet formation by modulating other processes that control axillary meristem development. These processes may involve auxin and gibberellin or sugars such as sucrose and trehalose, which influence inflorescence branching and are controlled by TB1-regulated genes in maize tiller buds [e.g., (*15*, *55*–*57*)]. Together with the localization of *HB-2* expression to spikelet primordia and the identification of DEGs that influence lateral organ development (e.g., *BAF1*, *SCL6*, and *PID*/*BIF2*), our results suggest that higher *HB-2* expression may promote paired spikelet development by enhancing the potential of the axillary meristem to form a short branch composed of two spikelets rather than a single spikelet. This role for *HB-2* would be consistent with the reported functions of *REV* in controlling axillary meristem initiation of *Arabidopsis* (*19*) and, as indicated by analyses of *ps1* and *ps2* stems and leaves, may involve modified vascular development or increased adaxialization of the spikelet primordium.

The influence of *HB-2* up-regulation on spikelet and leaf development provides more information about the function of HD-ZIP III transcription factors in grasses. Phylogenetic analyses (Fig. 2) indicate that *REV* of *Arabidopsis* has been duplicated in grasses to generate *HB-1* and *HB-2*, which are each expressed in developing inflorescences of wheat and rice (Fig. 3 and fig. S10) (*17*, *22*). In rice, a mutation in the miR165/166 complementary site of *HB-1/LF1*, a paralog of *HB-2*, promotes development of a lateral floret, which forms within the sterile lemmas located between the terminal floret and the rudimentary glumes of the spikelet (*22*). Thus, in rice, mutation of the miR165/166 complementary site of *HB-1* promotes formation of a spikelet with two florets, which is similar but anatomically distinct from the paired spikelets observed in *ps1* and *ps2.* This difference may indicate unique roles for *HB-1* and *HB-2* in regulating floret and spikelet development, respectively, which is supported by *WKNOX/OSH1* not being up-regulated in *ps1* or *Ps1/ps1*, as it was in *lf1* (*22*). Alternatively, given that wheat spikelets are indeterminate while those of rice are determinate, it may suggest that the effect of higher *HB-1* and *HB-2* expression on inflorescence development is dependent on determinacy of the axillary meristems. Regarding leaf development, mutations in the miR165/166 complementary site of *HB-1*, *HB-3*, and *HB-5* of rice promote the formation of narrow rolled leaves with reduced abaxial identity, much like those formed by *ps1* (*17*, *23*). Similar narrow rolled leaves are produced by the *Rld1-O* maize line that contains a mutation in the miR165/166 complementary site of *Rld1*, an ortholog of *HB-1* (*21*). Together, these results indicate that the two grass paralogs of *REV* share a conserved role regulating the adaxialization of the leaf blade, although analysis of *ps2* indicates that this function is not conserved by all wheat homeologs.

Our analysis of field-grown *Ps1/ps1* and *ps1* showed that higher expression of *HB-2* is associated with the production of grain that contains more protein. Higher protein content is associated with increased hydraulic conductivity of the inflorescence and peduncle and greater supply of amino acids to rachis, which are most likely facilitated by the formation of extra vascular bundles in the stem. The observed increase in protein content is substantial because it is comparable to that provided by *Gpc-B1* (*Grain Protein Content-B1*), a major locus regulating protein levels in wheat grain. The effect of *Gpc-B1* is underpinned by an allele of a NAC (NAM, ATAF, and CUC) transcription factor (*NAM-B1*) from wild wheat, which increases protein content by accelerating senescence and improving nutrient remobilization to developing grain, relative to lines with the nonfunctional alleles of cultivated wheat (*58*, *59*). The *Ps1/ps1* and *ps1* plants flowered and senesced at the same time as wild-type siblings, indicating that expedited senescence is not the cause of higher grain protein content in these genotypes. Our results suggest that higher protein content was not a consequence of reduced productivity, as grain number per inflorescence and yield per plant were not significantly lower in *Ps1/ps1* and *ps1* relative to wild-type siblings. With a view toward breeding inbred cultivars with improved grain quality, we propose that further analysis of *HB-2* should include editing alternate bases in the miR165/166 complementary site of each homeolog to identify mutations that increase grain protein content without modifying plant architecture or reducing yield component traits (e.g., thousand grain weight); studies in *Arabidopsis* indicate that mutation of alternate nucleotides in the miR165/166 complementary site differentially affects degradation of the target transcript (*45*). Alternatively, the semidominant alleles may be useful for breeding hybrid lines with higher GPC, as heterozygous *Ps1/ps1* lines produce grain with more protein and form normal leaves that do not restrict flowering. In summary, the *HB-2* alleles identified here provide a previously uncharacterized genetic strategy to breed cultivars that form grain with more protein, which is crucial given that grain protein content is a major determinant of wheat’s nutritional and economic value.

In conclusion, we show that mutations in the miR165/166 complementary site of *HB-2* promote paired spikelet development and are associated with the production of grain with more protein. These findings support a central role for miR165/166 in regulating expression of *REV-like* genes, and the stem and leaf phenotypes highlight a conserved function for HD-ZIP III transcription factors in regulating vascular development and adaxial identity of leaves (*18*, *20*, *21*, *41*). Our results provide a previously unidentified understanding of the role that HD-ZIP III transcription factors perform during cereal inflorescence development and unique knowledge about genes that regulate spikelet architecture in wheat. The identification of these *HB-2* alleles emphasizes an emerging theme of breeding, where mutations that disrupt microRNA-mediated regulation of developmental genes can help improve yield-related traits of cereals (*47*, *48*, *60*–*62*).

## MATERIALS AND METHODS

### Plant material and growth conditions

Hexaploid wheat (*T. aestivum*) used in this study included the following genotypes: cv. Cadenza, Mace, Rockstar, and Sheriff, a hexaploid wheat ethyl methanesulfonate-induced TILLING population (cv. Cadenza; 1752 lines) (*8*); wild-type, *Ps1/ps1*, and *ps1* near-isogenic lines (NILs) derived from *CAD1290* (BC_1_F_1_, BC_1_F_2_, BC_2_F_1_, BC_2_F_2-4_, and BC_3_F_2-3_ generations); transgenic lines expressing the *HB-D2* allele from *ps1* using the rice *Actin* promoter [*pActin:HB-D2* (*ps1*)], generated in the cv. Cadenza (see details below); and wild-type, *Ps2/ps2*, and *ps2* NILs derived from *CAD1761* (BC_1_F_1_, BC_1_F_2_, BC_1_F_2_, BC_2_F_1_, and BC_2_F_2-3_ generations); *Ppd-D1a* NILs [cv. Paragon; see (*25*)], which were crossed to *Ps1/ps1* to generate *Ppd-D1a*:*Ps1/ps1* and *Ppd-D1a*:*ps1* photoperiod-insensitive lines; *Ppd-D1*:*Ps1/ps1* and *Ppd-D1*:*ps1* photoperiod-sensitive lines (F_2_ generation); and *Ps1/ps1*, *ps1*, and *ps2* genotypes in the cv. Mace, Rockstar, and Sheriff backgrounds (BC_1_F_1_ generation). The “wild-type” genotypes for both *CAD1290* and *CAD1761* were respective NILs derived from segregating populations that did not display paired spikelet or leaf phenotypes; these wild-type lines are therefore expected to contain similar background mutations as the heterozygous and homozygous mutant lines. For both *CAD1290* and *CAD1761*, we analyzed two independent families generated from separate crosses to cv. Cadenza.

All field experiments were conducted at Church Farm, John Innes Centre, Bawburgh, Norfolk, UK (52°37′46.3″N, 1°10′48.2″E) in 1-m^2^ plots. The genetic screen for paired spikelet–producing lines was performed in 2015. Analyses of GPC, grain amino acids, and grain morphology/yield traits for WT, *Ps1/ps1*, and *ps1* were performed using plants grown in 2019 and 2020. The phenotype analysis, amino acid measurements of the rachis, gene expression studies, and hydraulic conductance experiments were performed on plants grown in controlled growth chambers under long-day (16-hour light/8-hour dark) photoperiods at 300 μmol/m^2^ per second [using Plantastar 400-W HQI bulbs (Osram) and Maxim 60-W tungsten bulbs], with day and night temperatures of 20° and 15°C, respectively. Plants for the crosses, segregation analysis, and GPC measurements were grown in glasshouses under long-day (16-hour light/8-hour dark) photoperiods, with day and night temperatures of 20° and 15°C, respectively. The extralong daylength experiments were performed in glasshouses with the photoperiod set to 22-hour light/2-hour dark using Heliospectra light-emitting diode lights (Heliospectra, Sweden), with day and night temperatures of 20° and 15°C, respectively.

### Phenotypic analysis

For the genetic screen of the TILLING population, paired spikelets were scored per plot rather than on individual plants, as each line displayed a degree of genetic heterogeneity (*8*). Class I mutants were defined as those that displayed infertile secondary spikelets on multiple tillers of an individual plant and on 50 to 100% of plants in the plot. Class II mutants were defined as those that formed fertile and infertile secondary spikelets on all tillers of an individual plant and on 50 to 100% of plants in the plot. Rachis nodes (primary spikelets) and secondary spikelets were recorded for inflorescences of the main stem and first tiller, as described previously (*9*). Secondary spikelet distribution was determined as described previously (*9*) using inflorescences from the main stem. The rate of inflorescence development was determined for the *Ps1/ps1*, *ps1*, and *ps2* genotypes, relative to their respective wild-type siblings, by measuring inflorescence length at intervals defined by leaf emergence (leaf 4 to 7). Flowering time was determined for each genotype at the emergence of the inflorescence from the boot, and images of the primary and secondary spikelets were obtained using a Zeiss Stemi 508 microscope with an Axiocam camera (Zeiss SMT, Germany).

For the field experiments, rachis nodes (primary spikelets) and secondary spikelets were determined for the main stem and the first two tillers of each plant, and both fertile and infertile secondary spikelets were included. Tiller number, height and plant dry weight were measured at maturity. Plants used for these analyses were harvested from the central row of each plot. Heading date, representing flowering time, was recorded as days after sowing when 50% of the spikes of a plot had emerged to 50% from the flag leaf sheath. Senescence was scored when the peduncle tissue of 75% of plants of a plot had transitioned from being green to yellow. Grain morphometric measurements (grain width, length, area, and thousand grain weight) were recorded using the MARVIN grain analyzer (GTA Sensorik GmbH, Germany). Measurements for each genotype include eight biological replicates, and each sample is composed of 150 to 250 grains. Harvest index was determined by dividing the weight of grain produced per plant by its above-ground biomass (dry weight).

### Scanning electron microscopy and light microscopy

Developing inflorescences at the floret primordium and terminal spikelet stages from wild-type, *Ps1/ps1*, *ps1*, and *ps2* were chemically fixed in 2.5% glutaraldehyde in 0.05 M sodium cacodylate (pH 7.4) overnight at 4°C. After rinsing, inflorescence samples were dehydrated through an ethanol series for 30 min each in 30, 50, 70, 90, 100, and 100% dry ethanol and then critical point dried using a Leica EM CPD300 (Leica Microsystems Ltd., UK). Dried samples were mounted on the surface of an aluminum pin stub using double-sided adhesive carbon discs (Agar Scientific Ltd., UK). The stubs were sputter-coated with approximately 15-nm gold in a high-resolution sputter coater (Agar Scientific Ltd.) and transferred to a Zeiss Supra 55 VP FEG scanning electron microscope. The samples were viewed at 3 kV.

For analysis of leaf cross sections, tissue was harvested from wild type, *Ps1/ps1*, and *ps1* at the double-ridge stage. Tissue sections were cut into 1-mm^2^ pieces and fixed in 2.5% (v/v) glutaraldehyde in 0.05 M sodium cacodylate (pH 7.3) overnight at room temperature. Fixative was removed by three successive washes in 0.05 M sodium cacodylate (15 min) and postfixed in 1% (w/v) osmium tetroxide in 0.05 M sodium cacodylate at room temperature for 2 hours. Samples were washed in distilled water (3 × 15 min) and were dehydrated in an ethanol series (30%, 50%, 70%, 95%, and two changes of 100% ethanol, each for an hour). Once dehydrated, the samples were gradually infiltrated with an LR White resin (London Resin Company, UK) by successive changes of resin:ethanol mixes at room temperature (1:1 for 1 hour, 2:1 for 1 hour, 3:1 for 1 hour, 100% resin for 1 hour, 100% resin for 16 hours, and 100% resin for 8 hours). The samples were transferred into gelatin capsules filled with fresh LR White and placed at 60°C for 16 hours to polymerize. Samples were sectioned using a Leica UC6 ultramicrotome, and semithin sections of 500 nm were stained with 0.5% Safranin-O (w/v) in 25% ethanol.

For stem cross sections of *Ps1/ps1*, *ps1*, *ps2*, and the wild-type siblings, tissue was harvested from the main stem subtending the mature inflorescence used for hydraulic conductance assays. The harvested stem tissue was fixed in 70% ethanol and was replaced every hour for three washes, and samples were stored at 4°C overnight. Samples were washed in distilled water (3 × 5 min), embedded in 4% agarose (w/v), and sectioned using a vibratome (Leica Microsystems Ltd.) into 70- to 100-μm sections. Sections were stained using 0.5% (w/v) toluidine blue in distilled water and imaged on a Nikon Ni-E microscope (Nikon, Japan). The number of vascular bundles was determined for three to four technical replicates for each biological replicate.

### DNA extractions and exome capture

Genomic DNA was extracted from leaves, as described previously (*10*). For the exome capture analysis of *CAD1290*, three bulks were generated using DNA from individuals of two independent BC_1_F_2_-segregating families, determined using inflorescence and leaf architecture phenotypes. The wild-type bulk included DNA of individuals with normal inflorescence and leaf architecture. The *Ps1/ps1* bulk included DNA of individuals with paired spikelets that formed normal leaves, and the *ps1* bulk contained DNA of plants with paired spikelets and curled leaves. For the exome capture of *CAD1761*, two bulks were generated using DNA from individuals of a BC_2_F_2_-segregating population. The first bulk included DNA of individuals with wild-type inflorescences, and the second bulk included DNA of paired spikelet–producing inflorescences.

Exome capture sequence analysis and data processing were performed as described previously (*63*), with the following modifications to facilitate SNP (single-nucleotide polymorphism) identification. SNPs were filtered for EMS-type mutations, i.e., G > A or C > T, and only mutations that had been predicted previously were considered for the subsequent analysis (*8*), as these were high-confidence SNPs likely to represent true mutations. *Ps1/ps1* and *ps1* bulks were filtered for either heterozygous or homozygous mutations, as well as mutations common between the bulks (i.e., if a heterozygous mutation existed in *Ps1/ps1*, then the corresponding mutation needed to be homozygous in *ps1*). By applying these filters, we identified 15 candidate mutations (table S9). Kompetitive allele–specific PCR (KASP)–based marker analysis was performed to test the segregation of these mutations and their association with the leaf and spikelet phenotypes in two separate BC_2_F_2_ populations and, later, in BC_3_F_2-3_ families. Of these mutations, Cadenza1290.chr1D.188459101 in *TraesCS1D02G137900* on chromosome 1D segregated with the phenotype(s). Next, all mutations identified for *CAD1290* in a 79.5-Mb region surrounding *TraesCS1D02G137900* were examined, and 16 were excluded a priori for being intron or silent mutations (table S10). KASP-based assays were performed on BC_3_F_2_ families to identify recombinant lines that segregated for mutations in this region and to identify alleles that uniquely associated with the leaf and spikelet phenotypes. A mutation that showed complete association with the phenotypes was identified in *TraesCS1D02G155200* (Cadenza1290.chr1D.217644011); this mutation displayed absolute heterozygosity with *Ps1/ps1* and homozygosity in *ps1* individuals for all independent families and generations. The same approach was used for exome capture analysis of the *CAD1761* DNA samples, with the exception that SNPs were filtered for the alternate allele in the paired spikelet–producing bulk (frequency, >0.8) and the reference allele in the wild-type bulk.

### Wheat transformation

The construct used to generate *pOsActin:HB-D2* transgenic plants contained the *HB-D2* coding sequence with the G575A mutation within the miR165/166 complementary site, as identified in *CAD1290/ps1*. The *OsActin* promoter was used to provide robust, but not overly strong, expression of the transgene; we did not attempt to use the native promoter because the genome sequence was poorly defined when the transgenics were generated nor did we use the *ZmUbi* promoter because it drives extreme overexpression in wheat and we were concerned that this would prevent the regeneration of transgenic seedlings given the role of *HB-2* on leaf adaxialization. The *pOsActin:HB-D2* construct was cloned into the L1P1 acceptor plasmid *pICH 47732* that contained the *Nos* terminator in a digestion-ligation reaction using Bsa I and T4 ligase. A second digestion-ligation reaction with Bpi I and T4 ligase cloned the cassette into the L2 acceptor pEC60606 L2V-GW. Transformation of immature embryos isolated from cv. Cadenza was performed by cocultivation with *Agrobacterium*, as described previously (*64*, *65*), by the Crop Transformation Group at National Institute of Agricultural Botany (Cambridge, UK). Presence of the transgene was confirmed by sequence analysis of the inserted copy of *HB-D2*, and the copy number of the transgene was determined using quantitative real-time PCR. The described leaf and inflorescence architecture traits (Fig. 2 and fig. S5) are from T_0_ and T_1_ generation plants, which are compared to null transgenic lines. The analyzed T_1_ generation plants all contained one copy of the transgene.

### KASP marker analysis and Sanger sequencing

Oligonucleotides for KASP analysis contained the standard FAM or HEX compatible tails (FAM tail, 5′-GAAGGTGACCAAGTTCATGCT-3′; HEX tail, 5′-GAAGGTCGGAGTCAACGGATT-3′). Oligonucleotide sequences for the KASP analyses of the mutations identified from the exome capture and those used to examine chromosome 1D mutations are provided in tables S13 and S14, respectively. The KASP assay was performed as described previously (*10*). DNA fragments were amplified by PCR, using either Phusion DNA polymerase (NEB, USA) or Q5 DNA polymerase (NEB), using oligonucleotides provided in table S15. PCR amplicons were sequenced using the Mix2Seq kit (Eurofins, Germany).

### RNA extractions and qRT-PCR

RNA was extracted from the following tissue types for the qRT-PCR analysis: young emerging leaves (lamina; harvested at glume primordium stage); mature leaves (lamina; harvested at white anther stage) with a developed ligule; flag leaf (lamina; harvested after ear emergence); stem nodes 1 to 3 before elongation of the internodes (nodes are numbered from apex to base, harvested before stem elongation); peduncle; whole developing inflorescences at the vegetative, double-ridge, glume primordium, and terminal spikelet stages; and pre-booting inflorescence. Samples for stem nodes and developing inflorescences included pooled samples from four to eight plants. RNA was extracted from the leaf, peduncle, and stem tissue using the Spectrum Plant Total RNA Kit (Sigma-Aldrich, USA), as well as from inflorescences at early and late developmental stages using the RNeasy Plant Mini Kit (QIAGEN, the Netherlands). Total RNA was treated with Turbo DNase I (Thermo Fischer Scientific, USA) before cDNA synthesis. Synthesis of cDNA and qRT-PCR were performed as described previously (*10*). Oligonucleotides for qRT-PCR analysis are provided in table S15. Expression of candidate genes was normalized using *TraesCS6D02G145100* and *TraesCS5A02G015600* [as described in (*10*)], and all data points are the average of at least three biological replicates and two technical replicates.

For RNA-seq analysis, RNA was extracted from whole inflorescences at the glume primordium stage, which was selected because this stage is crucial for primary and secondary spikelet formation (*9*) and it precedes floret primordia development, thus reducing tissue heterogeneity. For each sample, we pooled four to eight inflorescences, and three biological replicates were collected per genotype. Total RNA was extracted using the RNeasy Plant Mini Kit (QIAGEN) and treated with Turbo DNase I (Thermo Fisher Scientific). RNA was examined by gel electrophoresis; RNA purity was checked using the NanoPhotometer spectrophotometer (IMPLEN, USA), and RNA integrity was examined using the RNA Nano 6000 Assay Kit of the Bioanalyzer 2100 system (Agilent Technologies, USA). Library construction and RNA-seq were performed by Novogene (Novogene HK Company Ltd., Hong Kong). Sequencing libraries were generated using the NEBNext Ultra RNA Library Prep Kit for Illumina (NEB), and index codes were added to attribute sequences to each sample. For sequencing, clustering of the index-coded samples was performed on the cBot Cluster Generation System using the TruSeq PE Cluster Kit v3-cBot-HS (Illumina, USA). After cluster generation, the libraries were sequenced on an Illumina NovaSeq platform to generate 150-bp paired-end reads.

### In situ PCR analysis

In situ PCR analysis was performed as described previously (*66*) using 50 to 70 μm of cross sections of tissue prepared using a vibratome (Leica Microsystems Ltd.). Developing inflorescences were harvested from cv. Cadenza and *ps1* at the glume primordium and terminal spikelet stages, and stem tissue was collected from the node subtending the developing inflorescence at the glume primordium stage. These stages were selected on the basis of the qRT-PCR analysis (Fig. 3A) and because they define stages when primary and secondary spikelets initiate and secondary spikelets are visible. The oligonucleotide sequences used for first-strand cDNA synthesis and gene-specific PCR are provided in table S15. Sections were imaged on a Nikon Ni-E microscope.

### RNA-seq transcriptome analysis

Reads were aligned to the International Wheat Genome Sequencing Consortium (IWGSC) Chinese Spring gene model index v1.1 (*5*). Read alignment and expression quantification were performed using kallisto-0.42.3, as described previously (*49*). Differential gene expression analysis was achieved using the R program Sleuth v0.30.0, as described previously (*67*). Transcripts with a false discovery rate (FDR) adjusted *P* value (*q* value) < 0.05 were considered to be differentially expressed, and transcripts with a mean abundance of < 0.5 TPM in all three genotypes were excluded from further analysis. Differentially expressed transcripts were categorized into lists of up- and down-regulated genes in *Ps1/ps1* and *ps1*, relative to wild type for each pairwise comparison, on the basis of *q* value < 0.05 and mean TPM fold change > 0.5.

GO term enrichment analysis of up- and down-regulated transcripts was conducted using the R package GOseq v.1.40.0, as described previously (*67*, *68*). The gene universe was determined by genes with TPM > 0.5 for all genotypes. Significantly enriched GO terms were those that had adjusted *P* values of <0.05. Noteworthy significant biological process–related GO terms were identified using the web server REVIGO (*69*). Putative gene names of transcripts were determined on the basis of ortholog gene names in *A. thaliana* and *Zea mays* in Ensembl Plants BioMart (*70*).

For data visualization, normalized mean TPM (on a 0 to 1 scale) of each gene for all genotypes were visualized as a heatmap using the heatmap.2 function in the R package gplots v.3.0.4, where the distance measure is computed using Euclidean distance and hierarchical clustering was calculated via Ward’s minimum variance method (ward.D2). Genes of interest were selected by combining information from GO term enrichment, which were orthologs of *Arabidopsis* and maize genes with known roles during inflorescence development, and included two or three homeologs. These genes were visualized via heatmaps of relative TPM of *Ps1/ps1* and *ps1*, relative to wild type using the R package ggplot v.3.3.2 (*71*).

### Grain protein content analysis

Grain protein content measurements were performed on ca. 35 g of samples, using a DA 7250 near-infrared (NIR) spectrometer (Perten, Sweden). The NIR spectrometer was calibrated for protein measurements using a highly diversified set of grain that had been analyzed by the inductively coupled plasma mass spectrometry gold standard method. Four biological replicates were analyzed per genotype and condition.

### Hydraulic conductance experiments

Peduncle and mature inflorescences were harvested from the main stem of *Ps1/ps1*, *ps1*, *ps2*, and their respective wild-type siblings, at the early to medium milk stage when developing grain had filled the primary floret of central spikelets; all samples were measured between 6 and 8 hours after lights were turned on. Measurements of hydraulic conductance were performed immediately after sampling using a high-pressure flow meter (HPFM-Gen3, Dynamax, Houston, TX) in the transient state of operation. The HPFM was connected to the peduncle, which was recut under water at 3 cm below the base of the inflorescence. Increasing pressure from 0 to 500 kPa was applied to the peduncle, and flow rate was recorded every 2 s; all measurements were corrected to values at 25°C; three to four technical replicates were recorded for each sample. Hydraulic conductance was calculated from the slope of the plot of water flow versus the applied pressure, and values were normalized for the length of the sample.

### Analysis of amino acid levels

For analysis of free amino acids in grain, metabolites were extracted from flour aliquots (30 mg) in triplicate in D_2_O:CD_3_OD [1 ml; 4:1 containing 0.01% (w/v) d_4_-3-(trimethylsilyl) propionic acid (d_4_-TSP)] at 50°C for 10 min. After cooling and centrifugation (5 min), the supernatant was removed to a clean tube and heated (90°C, 2 min) to stop the residual enzyme activity. After cooling and centrifugation, 650 μl was transferred to a 5-mm nuclear magnetic resonance (NMR) tube for analysis. ^1^H-NMR spectra were collected at 300°K using the Avance Neo Spectrometer (Bruker BioSpin, UK) operating at 600.0528 MHz, equipped with a 5-mm triple resonance inverse (TCI) cryoprobe. Spectra were collected using the ZGPR water suppression pulse sequence with a 90° pulse and a relaxation delay of 5 s. Each spectrum was acquired using 16 scans of 65,536 data points with a spectral width of 7143 Hz. Spectra were Fourier-transformed using an exponential window with a line broadening value of 0.5 Hz. Phasing and baseline correction were carried out within the instrument software. ^1^H chemical shifts were referenced to d_4_-TSP at δ0.00. ^1^H-NMR spectra were automatically reduced, using AMIX (Analysis of MIXtures software, Bruker BioSpin), to ASCII files containing integrated regions or “buckets” of equal width (0.01 parts per million). Spectral intensities were scaled to the d_4_-TSP region (δ, 0.05 to −0.05). Amino acids were quantified via integration of characteristic signals from authentic standards run under identical conditions using the known concentration of internal standard (d_4_-TSP). Analysis of variance (ANOVA) was conducted using Spotfire Analyst (version 7.11.1; TIBCO). Four biological replicates were analyzed per genotype.

For the analysis of amino acid content in the rachis, free amino acids were extracted from 80 to 100 mg of tissue with 1 ml of 0.1 M HCl in an ultrasonic ice bath for 10 min. The resulting homogenates were centrifuged twice for 10 min at 4°C and 16,400*g* to remove cell debris. Derivatization and determination of proteinogenic amino acid levels was carried out as described previously (*72*). Six biological replicates were analyzed per genotype and tissue.

### Phylogenetic analysis

Orthologs and paralogs of *HB-2* from wheat (*T. aestivum*), *A. thaliana*, maize (*Z. mays*), and rice (*Oryza sativa*) were obtained by BLAST (Basic Local Alignment Search Tool) analysis using Ensembl Plants. The list of wheat genes encoding HD-ZIP III transcription factors is outlined in table S11. The top 31 protein sequences were aligned using the MAFFT v.7.123b alignment algorithm using 4 GUIDANCE v.2.0, with 100 bootstrap replicates (*73*, *74*). Subsequent evolutionary analyses were conducted in MEGA5 (*75*) using the maximum likelihood algorithm with the following parameters: Jones-Taylor-Thornton (JTT) matrix-based model, gamma distributed rates, and partial deletions. Bootstrap values are based on 100 replicates for testing the significance at nodes.

### Northern blot analysis

Total RNA was extracted from developing inflorescences of wild type and *ps1* using the total nucleic acid isolation method, with minor modifications (*76*). After lysing the ground tissue in extraction buffer, lysates were extracted twice using phenol and phenol/chloroform/isoamyl alcohol, followed by a single extraction with 2.5 volumes of chloroform. The extracted RNA was precipitated using absolute ethanol and incubated at −20°C for 3 hours to enrich for small RNAs. Precipitated RNA was washed twice with 70% ethanol (v/v) and resuspended in nuclease-free water.

The 3′Digoxigenin-labeled DNA probes for U6 small nuclear RNA, miR166, and miR172 were synthesized (Integrated DNA Technologies, Coralville, USA). Northern blot analysis was performed using 10 μg of total RNA, as described previously (*77*). The samples were transferred to positively charged Nylon membranes. The blots were ultraviolet (UV)–cross-linked using a Stratalinker UV cross-linker (Stratagene, USA). The blots were cut into two halves at the xylene cyanol track. For the U6 and miR172 probes, blots were prehybridized at 43°C for 30 min in the DIG Easy Granule (Sigma-Aldrich) and hybridized with the DIG-labeled probe overnight at 43°C; for miR172 probe, the blots were then prehybridized at 50°C for 30 min in the DIG Easy Granule (Sigma-Aldrich) and then hybridized with the DIG-labeled probe overnight at 50°C. Low-stringency washes (2× SSC + 0.1% SDS) followed by high stringency washes (0.1× SSC + 0.1% SDS) were performed on blots probed with U6 and miR172 probes. Only low-stringency washes were performed on the miR166 probed blots. The hybridized probe was visualized according to the manufacturer’s instructions. The second half of the blot was stripped using 0.2 M NaOH containing 0.1% SDS for 5 min twice at 37°C before reprobing with the miR166 DIG-labeled probe.

### Statistical analysis

The data shown in the figures are means ± SEM. In boxplots, the box is bound by the lower and upper quartiles, the central bar represents the median, and whiskers indicate the minimum and maximum values of the biological replicates.

Pairwise differences between genotypes or treatments were analyzed using a two-tailed Student’s *t* test. For comparisons involving more than two genotypes, the normality of data was tested using the Shapiro-Wilk test. Statistical analyses involving normally distributed data were performed using one-way ANOVA and Tukey post hoc analysis. Where assumptions of normality could not be met, Kruskal-Wallis and Dunnett’s post hoc tests were used to compare the genotypes or treatments. All data analyses were carried out using R (v4.0.0).

### Abbreviations

The following provides a guide to the gene names and identities used here along with the respective mutant alleles identified in this study: *HB-2*, *HOMEOBOX DOMAIN-2* (TraesCS1A02G157500, TraesCS1B02G173900, and TraesCS1D02G155200); *ps1*, *paired spikelet1*, the *HB-D2* allele identified in *CAD1290*; and *ps2*, *paired spikelet2*, the *HB-A2* allele identified in *CAD1761*.

## Notes

View/request a protocol for this paper from *Bio-protocol*.
