# Supplementary material for: MicroRNA-resistant alleles of HOMEOBOX DOMAIN-2 modify inflorescence branching and increase grain protein content of wheat
Source: Sci Adv. 2022 May 11;8(19):eabn5907. doi: 10.1126/sciadv.abn5907 (PMC9094671; doi:10.1126/sciadv.abn5907)
Supplement: Supplementary file 1 — Figs. S1 to S13 Tables S1 to S15 [file sciadv.abn5907_sm.pdf]

Science Advances
AAAS

Supplementary Materials for

MicroRNA-resistant alleles of HOMEOBOX DOMAIN-2 modify inflorescence branching and increase grain protein content of wheat

Laura E. Dixon, Marianna Pasquariello, Roshani Badgami, Kara A. Levin, Gernot Poschet, Pei Qin Ng, Simon Orford, Noam Chayut, Nikolai M. Adamski, Jemima Brinton, James Simmonds, Burkhard Steuernagel, Iain R. Searle, Cristobal Uauy, Scott A. Boden*

*Corresponding author. Email: scott.boden@jic.ac.uk

Published 11 May 2022, *Sci. Adv.* **8**, eabn5907 (2022)
DOI: 10.1126/sciadv.abn5907

The PDF file includes:

Figs. S1 to S13
Tables S1 to S15
Legend for data file S1

Other Supplementary Material for this manuscript includes the following:

Data file S1

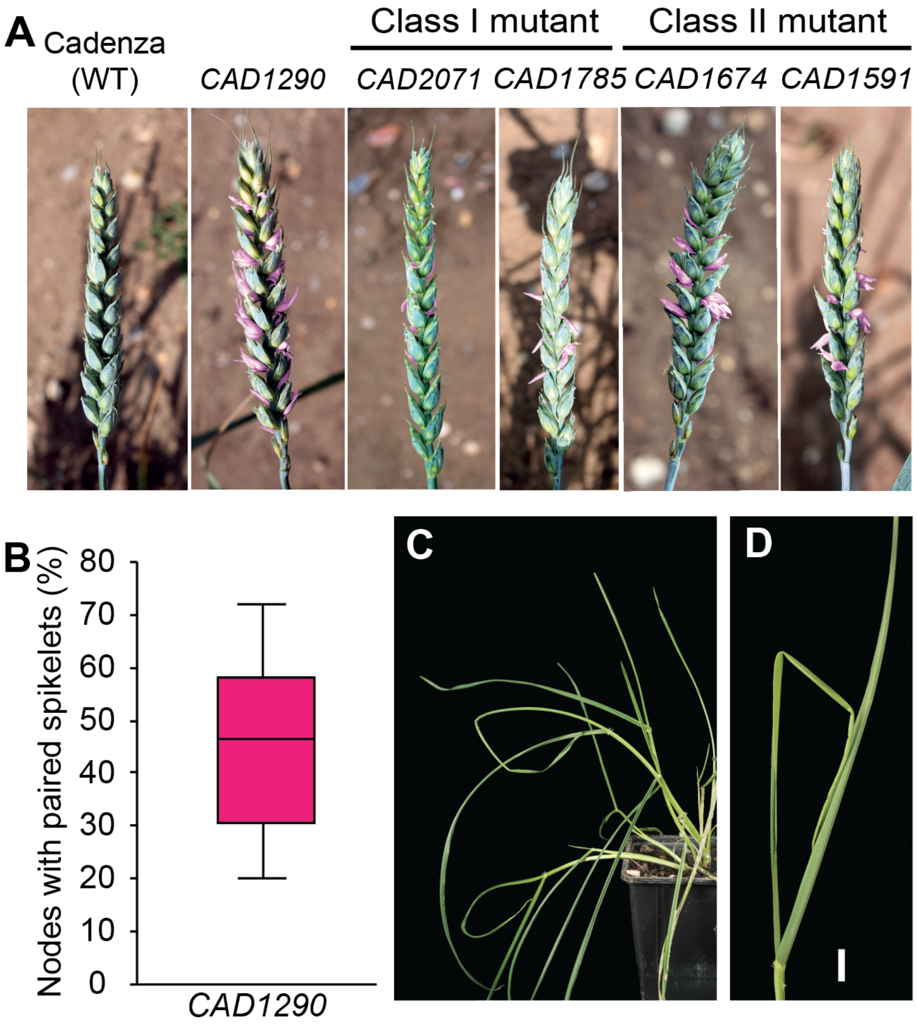

Fig. S1: Genetic screen of EMS-induced hexaploid wheat TILLING population identifies *CAD1290* as paired spikelet-producing line. (**A**) Representative inflorescences of wild-type (Cadenza), *CAD1290*, class I and class II paired spikelet-producing mutant lines. Secondary spikelets shown in pink. (**B**) Proportion of rachis nodes that form paired spikelets on *CAD1290* inflorescences, from plants grown under long-day controlled growth conditions. (**C-D**) Leaf phenotypes of *CAD1290* progeny, showing the leaf blade failing to emerge completely from the sheath. In the box-plot (**B**), the box is bound by the lower and upper quartiles, the central bar represents the median, and whiskers indicate minimum and maximum values of 20 biological replicates.

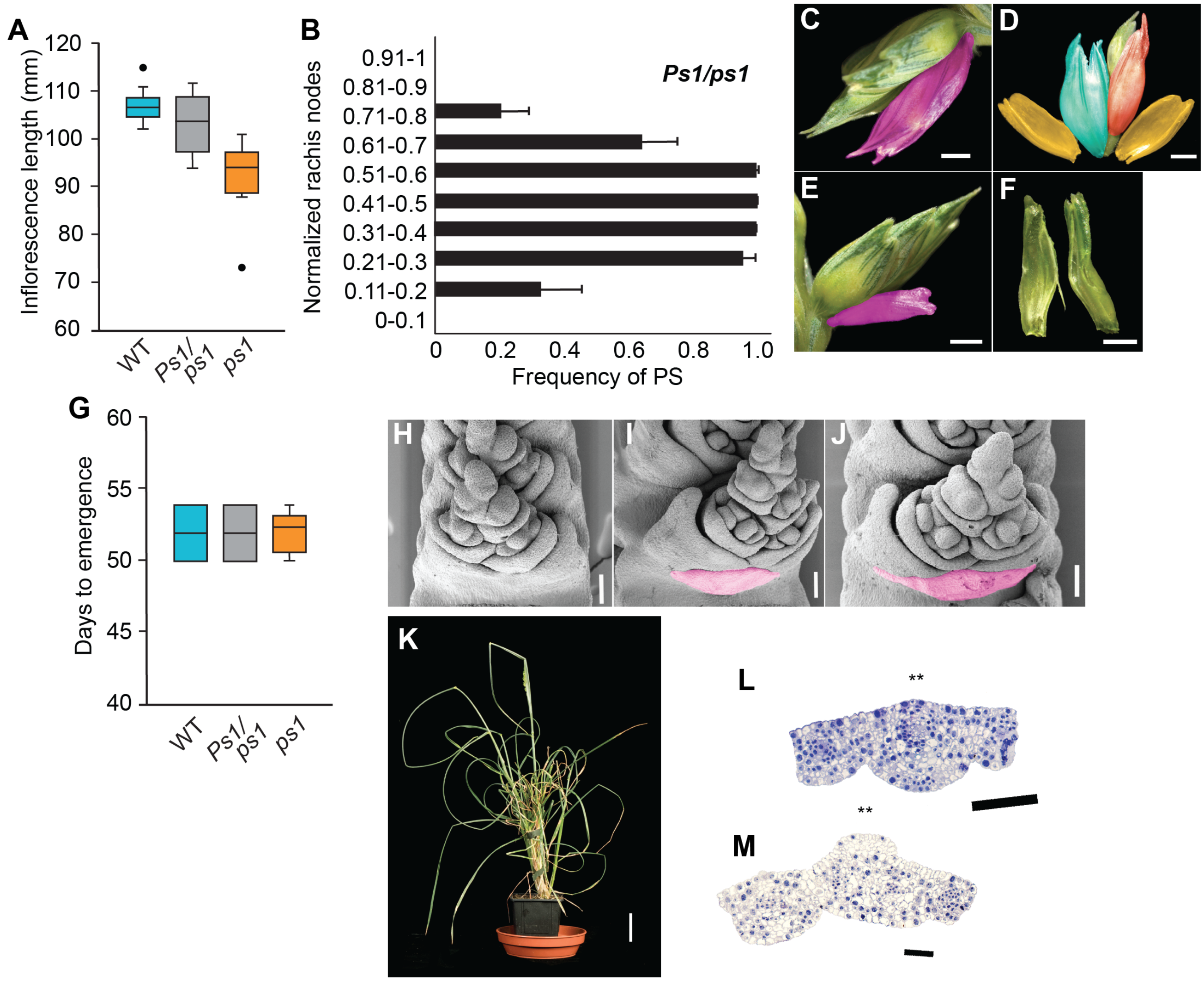

Fig. S2: Developmental phenotypes of *Ps1/ps1* and *ps1*. (**A**) Lengths of mature inflorescences from *Ps1/ps1* (grey) and *ps1* (orange), relative to wild-type siblings (blue). (**B**) The paired spikelets of *Ps1/ps1* plants form predominantly within the central region of the inflorescence. Bins 0-0.1 and 0.91-1 indicate the base and apex and the inflorescence, respectively. (**C**) A paired spikelet with a fertile secondary spikelet (pink), (**D**) showing the glume (yellow), primary (blue) and secondary (red) florets of the secondary spikelet. (**E**) A paired spikelet containing a rudimentary secondary spikelet (pink), (**F**) which does not contain florets with floral organs. (**G**) Flowering-time of glasshouse-grown *ps1* plants for which the leaves have been manually unfurled, relative to *Ps1/ps1* and wild-type, shown as days until inflorescence emergence. (**H-J**) Scanning electron micrographs of wild-type, *Ps1/ps1* and *ps1* inflorescences at the floret primordium stage – primary spikelets are in grey and secondary spikelets are highlighted in pink. (**K**) A *ps1* plant at inflorescence emergence, showing the curled flag leaf phenotype. (**L-M**) Toluidine blue-stained

cross sections of immature leaves from (**L**) WT and (**M**) *ps1*, showing abnormal mid-ribs (**). Scale bars, 1 cm (**C-F**); 100 µM (**H-J, L-M**); 5 cm (**K**). (**B**) Data are average ± of 8-10 biological replicates. In the box-plot (**A, G**), the box is bound by the lower and upper quartiles, the central bar represents the median, and whiskers indicate minimum and maximum values of 8-10 biological replicates.

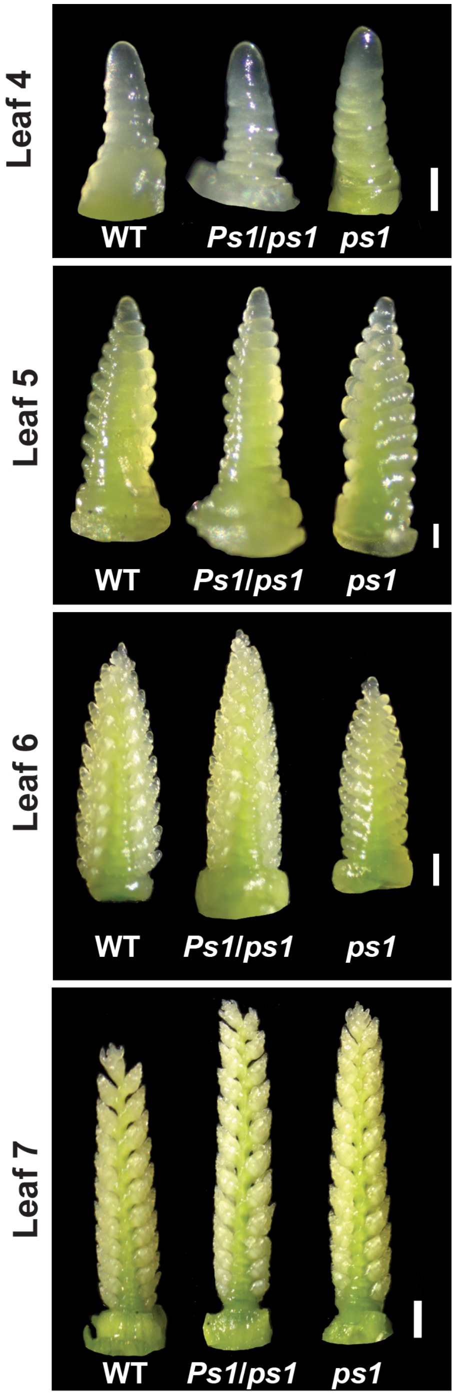

Fig. S3: Analysis of inflorescence development. Representative images of developing inflorescences from *Ps1/ps1* and *ps1* plants, relative to wild-type siblings, according to developmental stage determined by leaf number. Scale bars, (**L4-5**) 0.2 mm, (**L6**) 0.5 mm, (**L7**) 1 mm.

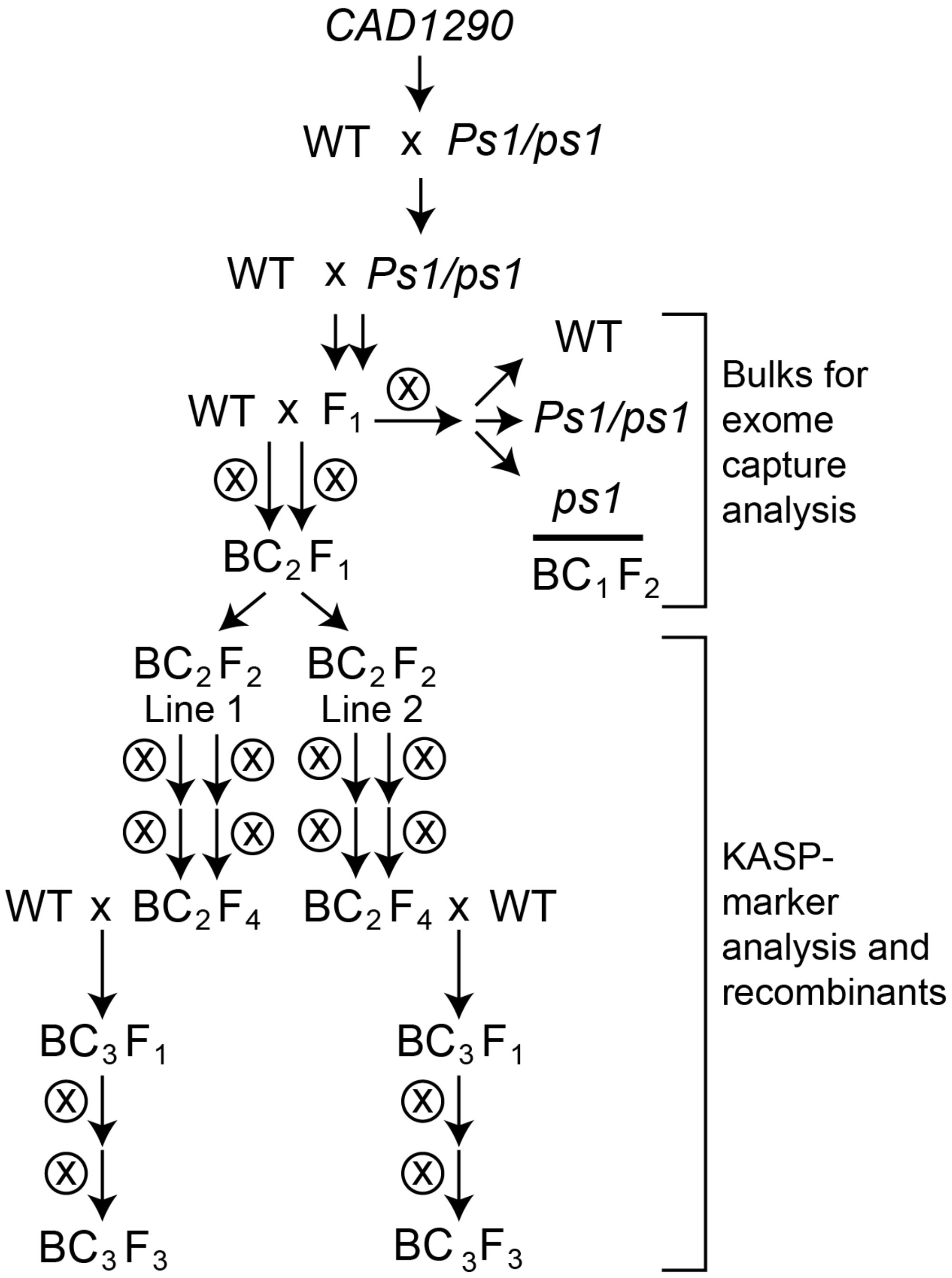

Fig. S4: Schematic of pedigree for genotypes used for genetic analysis of *CAD1290*. Progeny of a paired spikelet producing *CAD1290* plant was crossed to wild-type *cv.* Cadenza (WT) to generate back-crossed populations (BC), including first, second and third filial generations (F_1, F_2, F_3). Self-crosses are indicated by circled crosses.

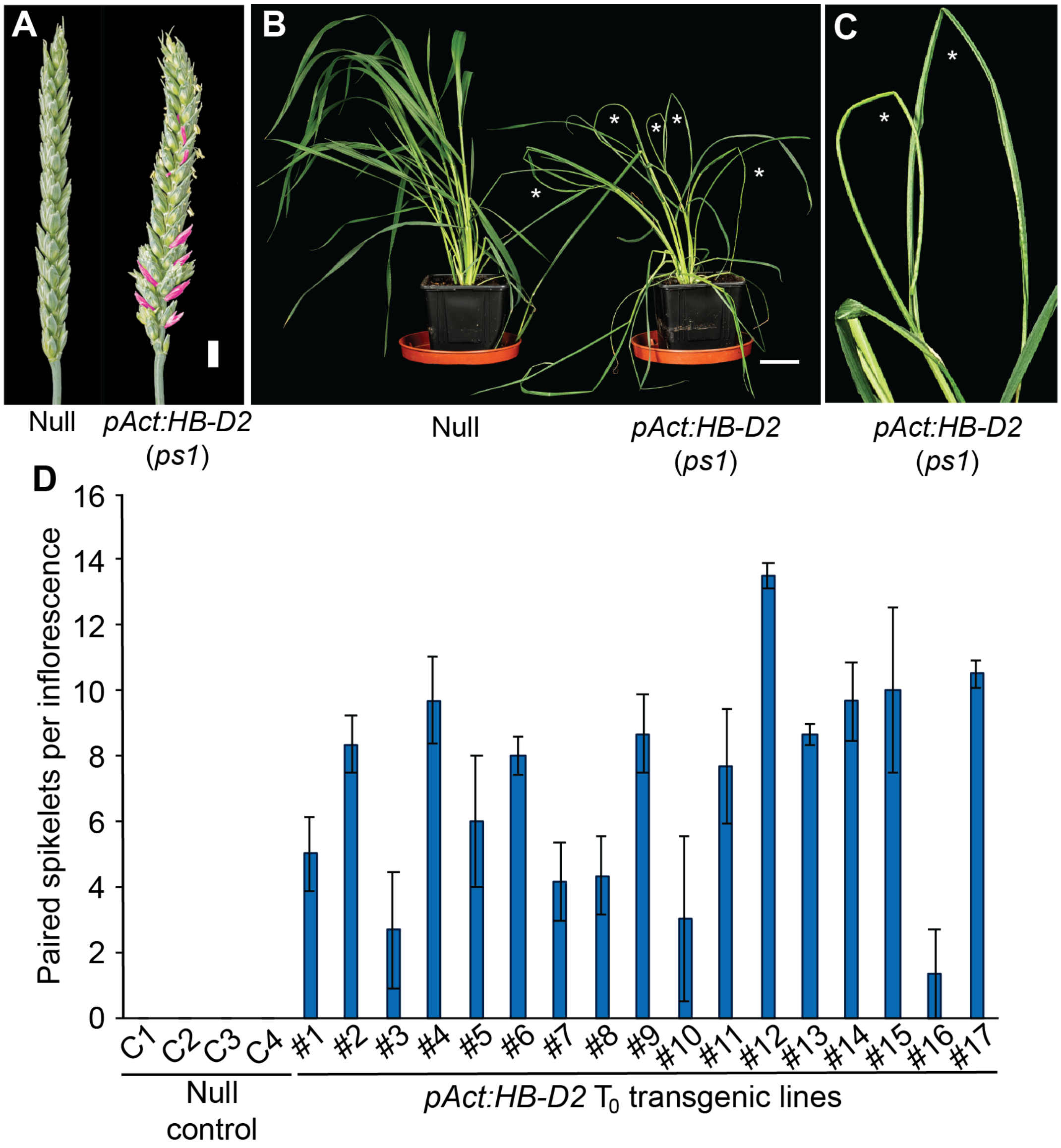

Fig. S5 – Transgenic *pActin:HB-D2* (*ps1*) plants produce paired spikelets and curled leaves. T_0 *pActin:HB-D2* (*ps1*) transgenic plants produce (**A**) inflorescences with paired spikelets (secondary spikelets shown in pink), and (**B-C**) curled leaves, with the leaf blade failing to emerge completely from the sheath (white asterisks) – null control plants do not show these phenotypes. (**D**) Independent T_0 *pActin:HB-D2* (*ps1*) transgenic plants form paired spikelets, relative to independent null transgenic control lines that form inflorescences without secondary spikelets. Data are the average ± s.e.m. of 2-3 inflorescences per plant. Scale bars, (**A**) 1 cm, (**B**) 5 cm.

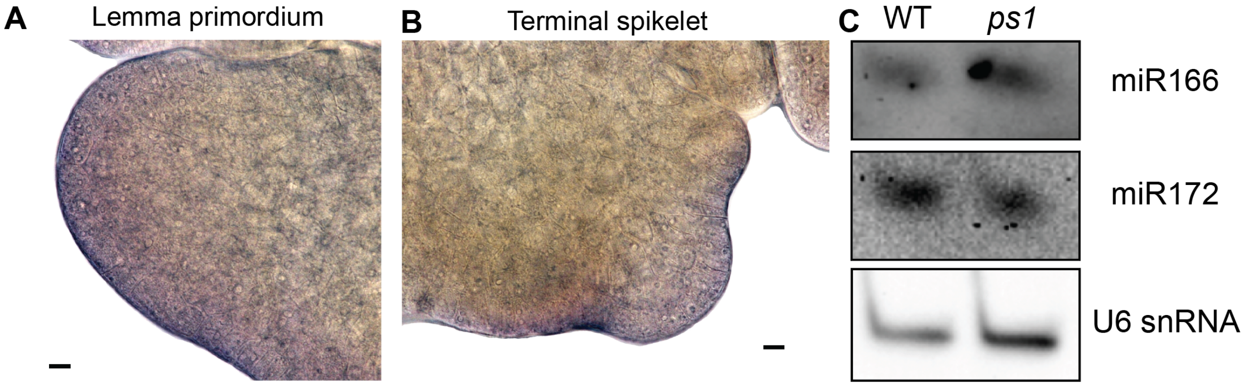

Fig. S6 – Analysis of *HB-2* and miR166 expression in developing inflorescences. (**A-B**) *HB-2* is expressed in spikelet primordia of *ps1* developing inflorescences at the (**A**) lemma primordium and (**B**) terminal spikelet developmental stages, as indicated by the purple staining (*HB-2* transcripts). (**C**) Developing inflorescences of wild-type and *ps1* express miR166. Loading controls include miR172 and U6 snRNA. Scale bars, (**A-B**) 10 μm.

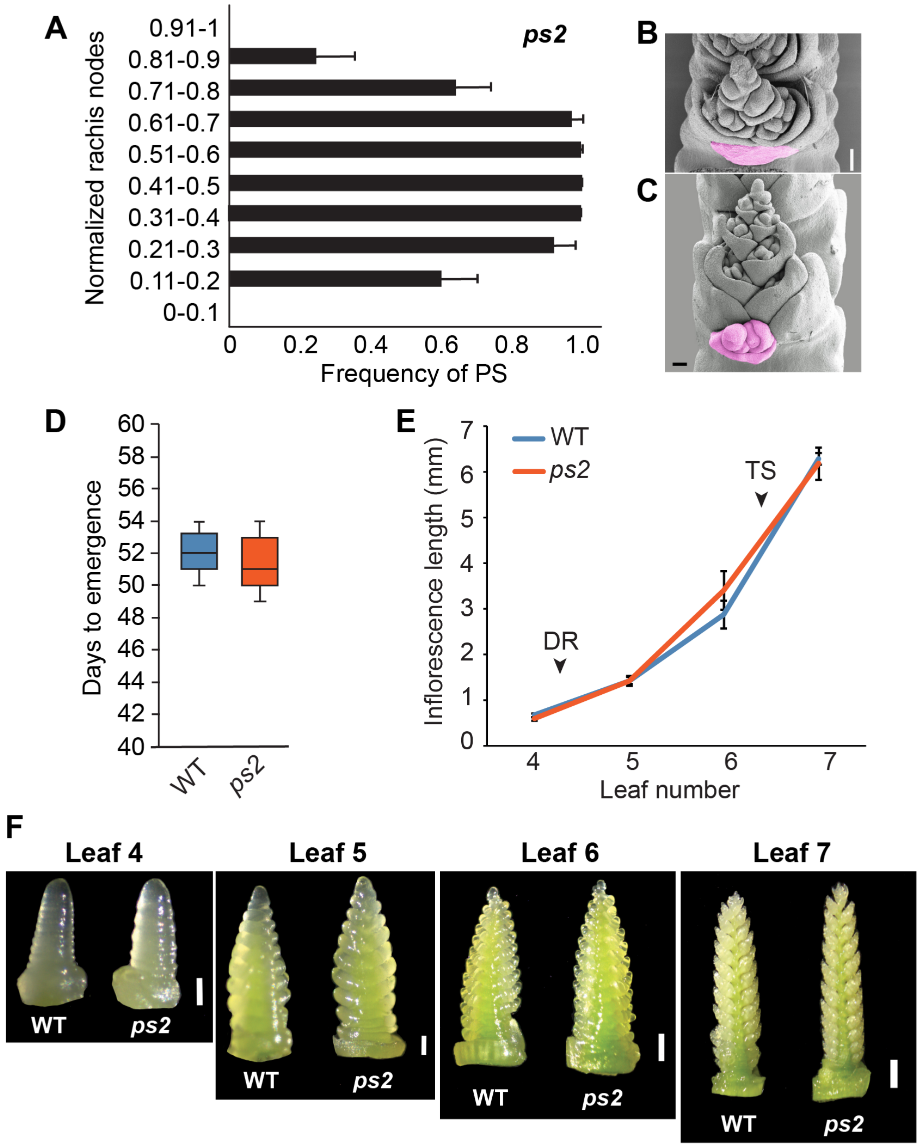

Fig. S7 – Developmental phenotypes of *ps2*. (**A**) The paired spikelets of *ps2* are distributed with the highest frequency in the middle of the inflorescence. Bins 0-0.1 and 0.91-1 represent the base and apex and the inflorescence, respectively. (**B-C**) Scanning electron micrographs of *ps2* inflorescences at the (**B**) floret primordium and (**C**) terminal spikelet stages – primary spikelets are shown in grey and secondary spikelets in pink. (**D**) Flowering-time of glasshouse-grown *ps2*, relative to wild-type siblings. (**E-F**) Analysis of inflorescence growth for *ps2*, relative to wild-type siblings, according to developmental stage determined by leaf number. Data are average ± s.e.m of (**A**) 10 and (**E**) 4-5 biological replicates. In the box-plot (**D**), the box is bound by the lower and upper quartiles, the central bar represents the median, and whiskers indicate minimum and maximum values of 10 biological replicates. Scale bars, (**A-B**) 0.2 mm, (**C**) 0.5 mm, (**D**) 1 mm.

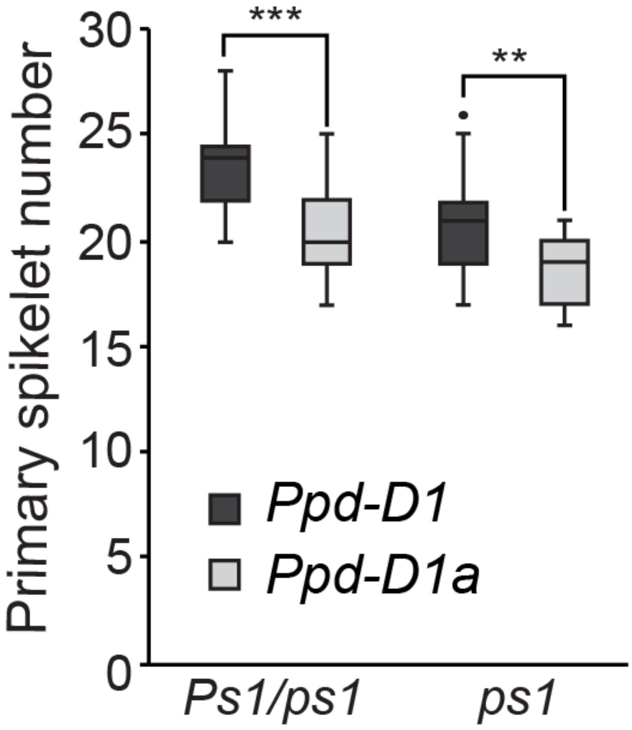

Fig. S8 – Analysis of spikelet numbers in photoperiod sensitive and insensitive lines. *Ps1/ps1* and *ps1* lines expressing the photoperiod insensitive *Ppd-D1a* allele form fewer primary spikelets than those with the photoperiod sensitive *Ppd-D1* allele. Each box is bound by the lower and upper quartiles, the central bar represents the median, and whiskers indicate minimum and maximum values of 12-24 biological replicates. ***$P < 0.001$.

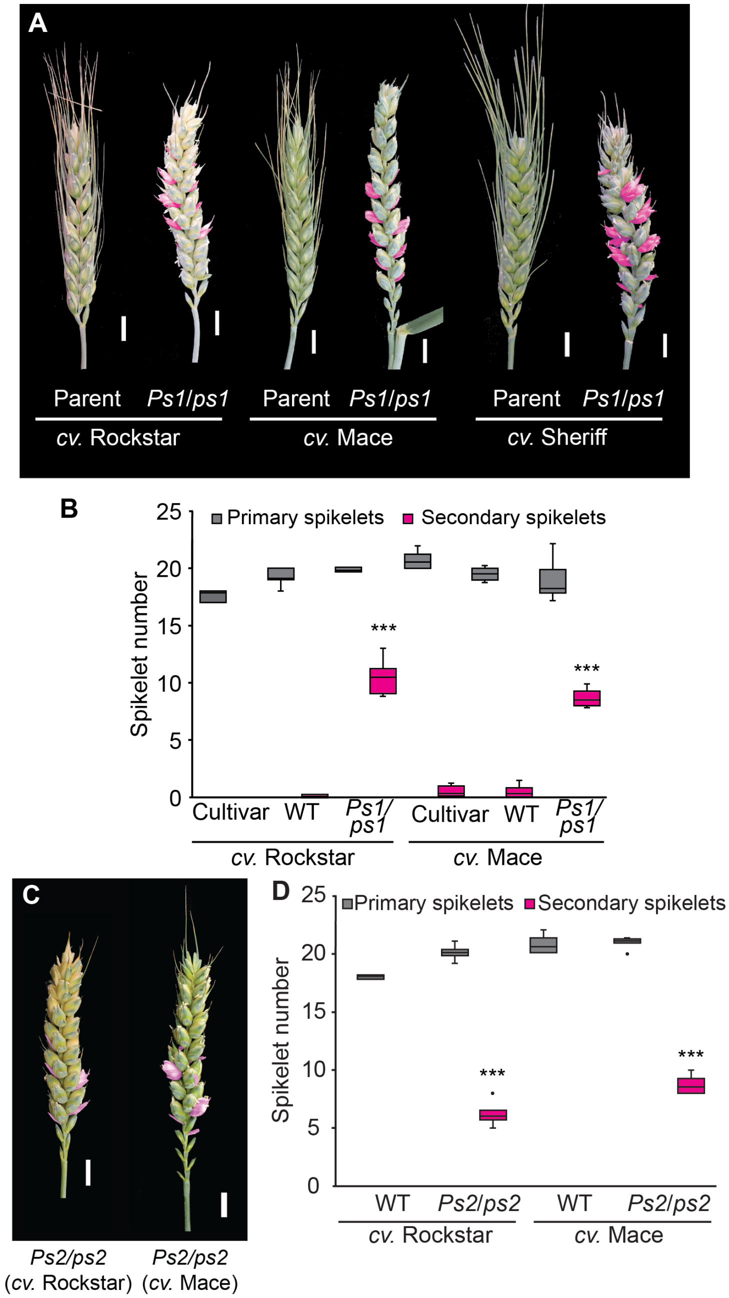

Fig. S9 – Spikelet architecture traits of elite cultivars that contain the *ps1* or *ps2* alleles of *HB-2*. Introduction of the *ps1* (**A-B**) or *ps2* (**C-D**) alleles of *HB-D2* and *HB-A2*, respectively, into elite cultivars (Rockstar, Mace and Sheriff) promotes secondary spikelet formation, relative to the cultivar and sibling lines with the wild-type allele of *HB-2*. Scale bars, 1 cm. Data are the average ± s.e.m. of 4-8 biological replicates. $^{***}P < 0.001$.

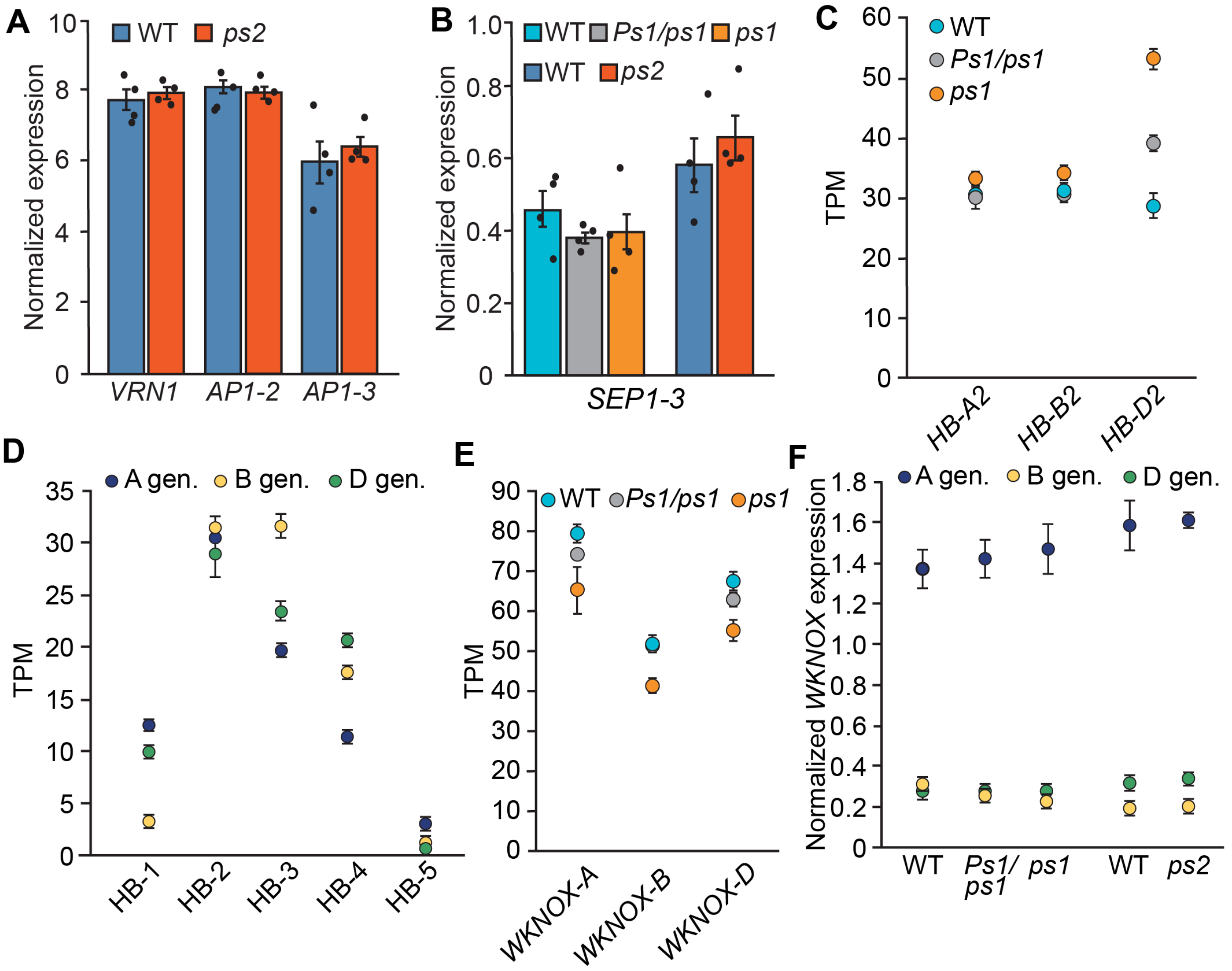

Fig. S10 – Gene expression analysis during development of *ps2* and *ps1* inflorescences. (**A**) Spikelet meristem identity genes are not differentially expressed in developing inflorescences of *ps2*, relative to wild-type siblings. (**B**) *SEP1-3* is not differentially expressed in developing inflorescences of *Ps1/ps1*, *ps1* or *ps2*, relative to their respective wild-type siblings (**C**) RNA-seq based quantification of *HB-A2*, *-B2* and *-D2* transcripts in developing inflorescences of *Ps1/ps1* and *ps1* plants, relative to wild-type siblings. (**D**) Transcript levels for each of the wheat genes encoding a class III homeodomain-leucine zipper transcription factor, as detected by RNA-seq analysis of developing inflorescences in wild-type – levels for the A (blue), B (yellow) and D (green) homeologs are shown. (**E-F**) RNA-seq (**E**) and qRT-PCR (**F**) based quantification of *WKNOX-A1*, *-B2* and *-D2* transcripts in developing inflorescences of *Ps1/ps1, ps1* and *ps2*, relative to wild-type siblings. Data are the average ± s.e.m. of (**A, B, F**) 4 or (**C-E**) 3 biological replicates. TPM, transcripts per million.

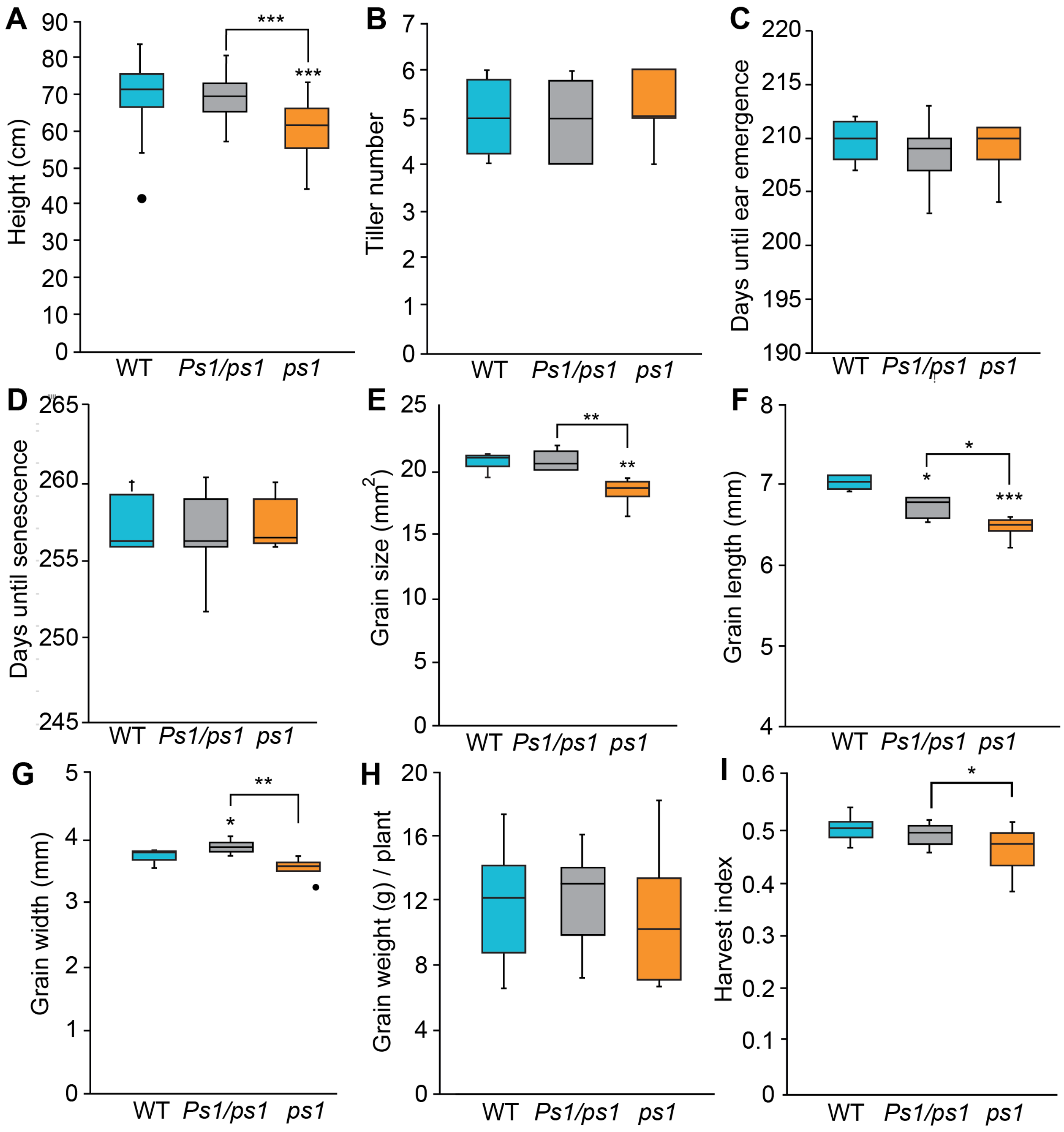

Fig. S11: Phenotypic analysis of field-grown *Ps1/ps1* and *ps1* plants. Quantification of (**A**) height, (**B**) tiller numbers, (**C**) flowering time, (**D**) timing of senescence, (**E**) grain size, (**F**) grain length, (**G**) grain width, (**H**) grain weight (grams) per plant, and (**I**) harvest index for *Ps1/ps1* and *ps1* plants grown in the field, relative to wild-type siblings. In the box-plots, the box is bound by the lower and upper quartiles, the central bar represents the median, and whiskers indicate minimum and maximum values of 20-24 biological replicates (**A-C**) or 6 biological replicates (**D-F**). * $P < 0.05$; ** $P < 0.01$; *** $P < 0.001$.

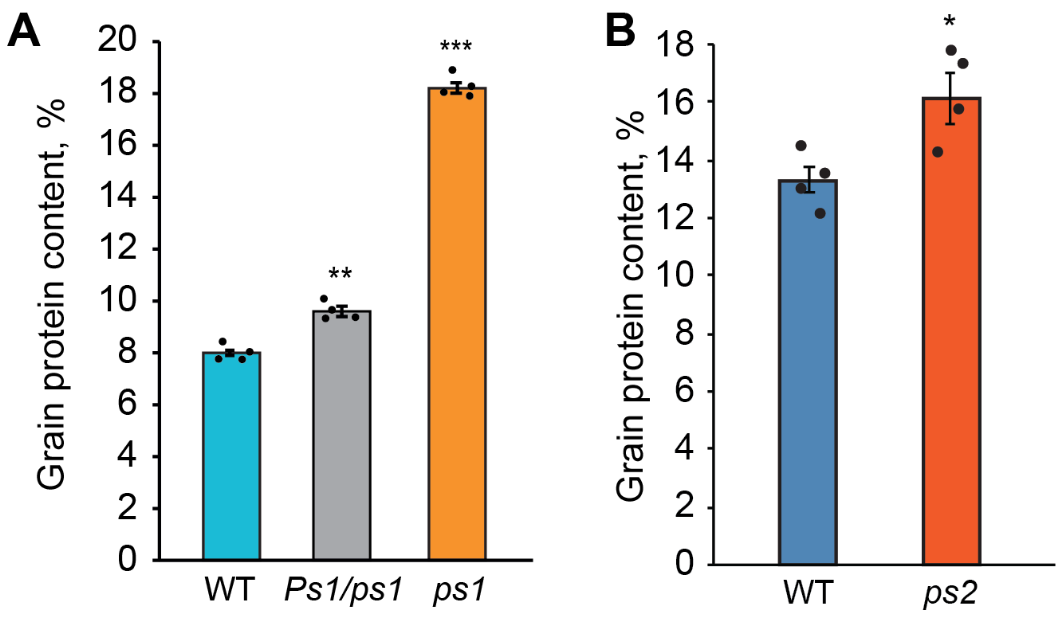

Fig. S12: Analysis of grain protein content in *Ps1/ps1*, *ps1* and *ps2* mutants. (**A**) Grain protein content (GPC) is higher in *Ps1/ps1* and *ps1*, relative to wild-type siblings, for glasshouse-grown plants. (**B**) GPC is higher in *ps2*, relative to wild-type siblings. Data are average ± s.e.m. of four biological replicates. * $P < 0.05$; ** $P < 0.01$; *** $P < 0.001$.

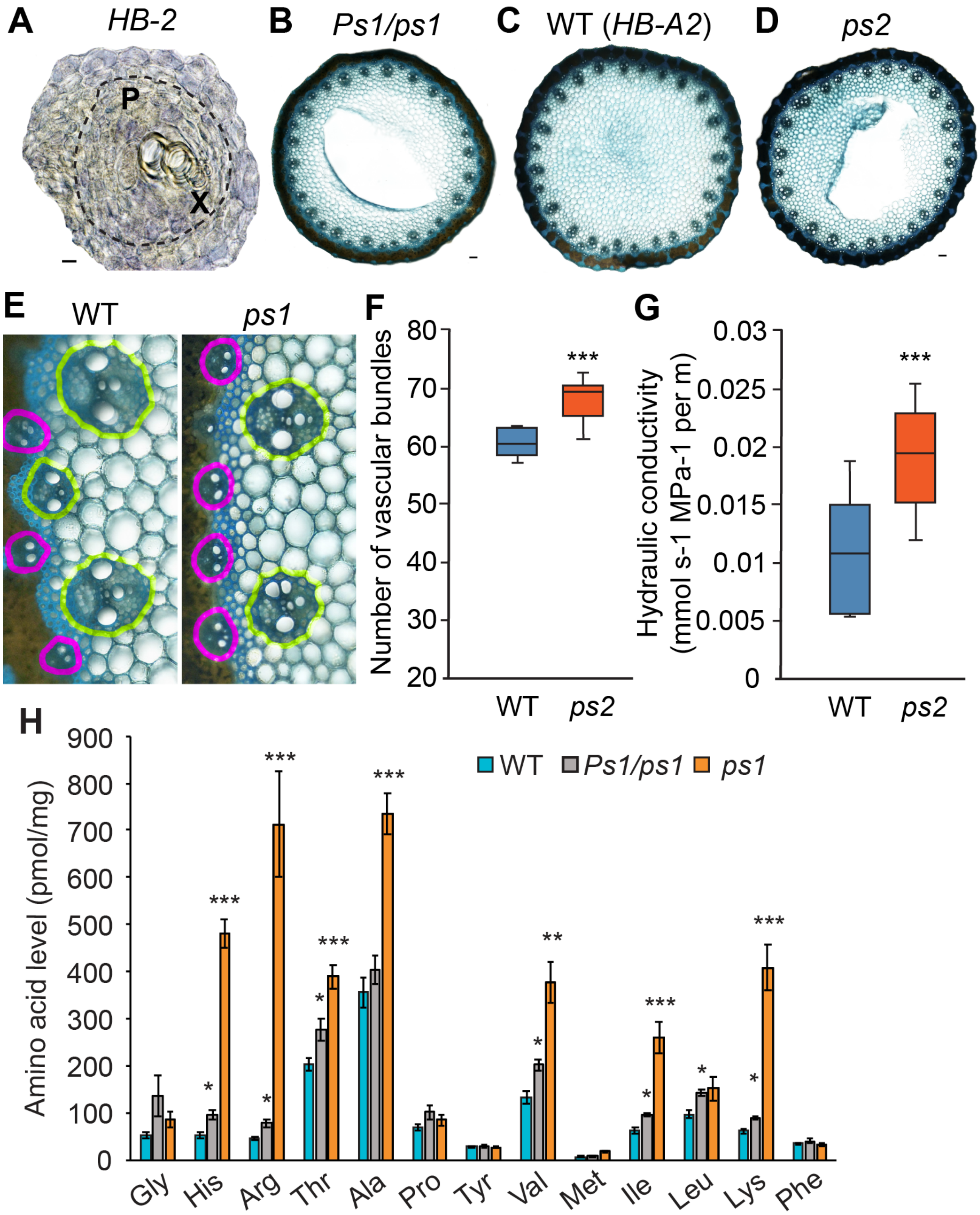

Fig. S13 – Analysis of plant vasculature and rachis amino acid content. (**A**) A replicate image of *in-situ* PCR analysis showing *HB-2* is expressed in vascular bundles of the stem, and in cells surrounding the vasculature. The dashed line indicates the boundary of the vascular bundle, with

xylem (X) and phloem (P) regions. (**B-D**) Toluidine blue stained cross-sections of peduncles from (**B**) *Ps1/ps1* heterozygous mutants, and (**C-D**) the *ps2* mutant (**D**), relative to its wild-type sibling (**C**). (**E-F**) Higher magnification images of stained peduncle cross sections from wild-type and *ps1* highlighting the two rings of vascular bundles of wheat stems; bundles of the inner and outer rings are highlighted in green and magenta, respectively. (**F**) Stems of *ps2* plants contain more vascular bundles than wild-type siblings. (**G**) Analysis of hydraulic conductivity in the peduncle and mature inflorescence of *ps2*, relative to its wild-type sibling. (**H**) Levels of amino acids are higher in rachises of *Ps1/ps1* and *ps1*, relative to wild-type siblings (WT). The amino acids shown are those not presented in Fig. 8. In the box-plots (**E-F**), the box is bound by the lower and upper quartiles, the central bar represents the median, and whiskers indicate minimum and maximum values of (**E**) 6-8 and (**F**) 10 biological replicates. (**G**) Data are the average ± s.e.m. of 6 biological replicates. Scale bars, (**A**) 10 μm, (**B, D**) 100 μm. *$P < 0.05$, ***$P < 0.001$.

Table S1: Summary of paired spikelet-producing TILLING line.

Phenotype	TILLING lines
**Class I:** moderate formation of paired spikelets, including rudimentary/infertile secondary spikelets, low numbers of PS per inflorescence and medium frequency of PS within plot.	CAD0016, CAD0021, CAD0030, CAD0039, CAD0040, CAD0042, CAD0045, CAD0063, CAD0071, CAD0073, CAD0074, CAD0082, CAD0087, CAD0096, CAD0097, CAD0109, CAD0142, CAD0143, CAD0144, CAD0149, CAD0166, CAD0167, CAD0174, CAD0177, CAD0183, CAD0189, CAD0191, CAD0193, CAD0197, CAD0200, CAD0205, CAD0208, CAD0226, CAD0227, CAD0236, CAD0246, CAD0247, CAD0248, CAD0266, CAD0268, CAD0273, CAD0274, CAD0278, CAD0297, CAD0299, CAD0302, CAD0313, CAD0319, CAD0326, CAD0328, CAD0334, CAD0338, CAD0351, CAD0355, CAD0361, CAD0373, CAD0375, CAD0426, CAD0434, CAD0435, CAD0445, CAD0448, CAD0456, CAD0461, CAD0463, CAD0471, CAD0481, CAD0488, CAD0495, CAD0498, CAD0502, CAD0504, CAD0520, CAD0529, CAD0551, CAD0557, CAD0558, CAD0568, CAD0571, CAD0589, CAD0601, CAD0608, CAD0626, CAD0631, CAD0636, CAD0638, CAD0665, CAD0668, CAD0688, CAD0708, CAD0723, CAD0725, CAD0731, CAD0736, CAD0737, CAD0759, CAD0763, CAD0773, CAD0776, CAD0784, CAD0825, CAD0829, CAD0880, CAD0906, CAD0934, CAD0953, CAD0964, CAD0972, CAD0977, CAD1024, CAD1025, CAD1028, CAD1029, CAD1049, CAD1059, CAD1063, CAD1073, CAD1084, CAD1088, CAD1092, CAD1112, CAD1116, CAD1118, CAD1126, CAD1140, CAD1153, CAD1171, CAD1176, CAD1181, CAD1182, CAD1183, CAD1186, CAD1187, CAD1188, CAD1189, CAD1198, CAD1214, CAD1215, CAD1221, CAD1247, CAD1257, CAD1262, CAD1263, CAD1270, CAD1272, CAD1287, CAD1308, CAD1313, CAD1324, CAD1349, CAD1354, CAD1364, CAD1374, CAD1399, CAD1400, CAD1414, CAD1436, CAD1441, CAD1498, CAD1523, CAD1561, CAD1563, CAD1566, CAD1569, CAD1570, CAD1601, CAD1606, CAD1628, CAD1634, CAD1639, CAD1648, CAD1651, CAD1653, CAD1658, CAD1661, CAD1663, CAD1672, CAD1677, CAD1682, CAD1694, CAD1695, CAD1701, CAD1703, CAD1705, CAD1713, CAD1717, CAD1726, CAD1730, CAD1735, CAD1758, CAD1761, CAD1775, CAD1785, CAD1809, CAD1839, CAD1850, CAD1856, CAD1982, CAD1986, CAD2001, CAD2023, CAD2071, CAD2084, CAD2090
**Class II:** robust and frequent formation of paired spikelets, including high number of PS per inflorescence, development of fertile secondary spikelets, and high frequency of PS within plot.	CAD0051, CAD0061, CAD0066, CAD0239, CAD0350, CAD0487, CAD0559, CAD0631, CAD0632, CAD0721, CAD0745, CAD0753, CAD0803, CAD0900, CAD0908, CAD0917, CAD0946, CAD0948, CAD0957, CAD0988, CAD1019, CAD1089, CAD1046, CAD1049, CAD1072, CAD1084, CAD1125, CAD1216, CAD1250, CAD1272, CAD1290, CAD1335, CAD1371, CAD1379, CAD1382, CAD1397, CAD1547, CAD1563, CAD1579, CAD1591, CAD1599, CAD1643, CAD1669, CAD1674, CAD1697, CAD1761, CAD1764, CAD1800, CAD1872, CAD1944, CAD2068, CAD2073

Table S2: Phenotypes of *CAD1290* progeny selected from field.

Line	Plants with WT inflorescences	Plants with paired spikelets	Total
CAD1290	6	6	12

NB: These data are from progeny of a single paired spikelet-producing plant of line *CAD1290*, which was selected from the screen of the Cadenza mutant population. These plants include #6 and #11 that were used for the backcrossing to *cv.* Cadenza (see Table S3-S8).

Table S3: Phenotypes of *CAD1290* lines used for backcrossing to Cadenza.

Line	Rachis nodes_1	PS_1	Rachis node_2	PS_2
#6	19	10	20	12
#11	19	13	19	11

NB: These data are from two inflorescences of the *CAD1290* plants used to perform backcrosses. Rachis nodes and number of paired spikelets (PS) per inflorescence are shown.

Table S4: Segregation ratios of F_1 generation of Cadenza × *Ps1/ps1*.

Line	WT	*Ps1/ps1*	*ps1*	Total
#6	3	3	-	6
#11	2	4	-	6

NB: These data show how many plants produced either WT or paired spikelet-producing inflorescences (*Ps1/ps1*) from the BC_1F_1 generation. The data are of the progeny derived from the first backcross to *cv.* Cadenza (Fig. S4).

Table S5: Segregation ratios of BC_1F_2 generation, progeny of *Ps1/ps1*.

Line	WT	*Ps1/ps1*	*ps1*	Total	χ^2
6.1-4	14	23	10	47	0.704
11.1-3	9	15	6	30	0.741

NB: These data show how many plants produced either WT, paired spikelet-producing inflorescences (*Ps1/ps1*) or paired spikelet-producing inflorescences with curled leaves from the BC_1F_2 generation.

Table S6: Segregation ratios of BC_2F_1 generation of Cadenza × *Ps1/ps1* (BC_1F_1).

Line	WT	*Ps1/ps1*	*ps1*	Total
6.1	8	4	-	12
6.4	8	0	-	8
6.5	5	2	-	7
6.6	1	1	-	2
11.1	3	5	-	8
11.2	2	0	-	2
11.3	3	1	-	4

NB: These data show how many plants produced either WT or paired spikelet-producing inflorescences (*Ps1/ps1*) from the BC_2F_1 generation. The data are of progeny derived from the second backcross to *cv.* Cadenza (Fig. S4).

Table S7: Segregation ratios of BC_2 generations, progeny of *Ps1/ps1*.

Generation	Line	WT	*Ps1/ps1*	*ps1*	Total	χ^2
BC_2F_2	6.6.1	27	45	22	94	0.704
BC_2F_2	11.3.4	25	46	22	93	0.904
BC_2F_3	6.6.1.11	27	45	22	94	0.704
BC_2F_3	6.6.1.60	25	46	22	93	0.904
BC_2F_3	11.3.4.33	27	45	22	94	0.704
BC_2F_3	11.3.4.35	27	45	22	94	0.704

NB: These data show how many plants produced either WT, paired spikelet-producing inflorescences (*Ps1/ps1*) or paired spikelet-producing inflorescences with curled leaves from the BC_2 generations.

Table S8: Segregation ratios of BC_3 generations, progeny of *Ps1/ps1*.

Generation	Line	WT	*Ps1/ps1*	*ps1*	Total	χ^2
BC_3F_2	3.5	26	48	18	92	0.457
BC_3F_2	4.4	23	47	23	93	0.995
BC_3F_3	3.5.24	23	48	22	93	0.943

NB: These data show how many plants produced either WT, paired spikelet-producing inflorescences (*Ps1/ps1*) or paired spikelet-producing inflorescences with curled leaves from the BC_3 generations.

Table S9: List of mutations identified in the exome capture analysis of *CAD1290*.

**Chr.**	**Cadenza scaffold***	**Scaffold position**	**Gene ID†**	**Mutation ID/position**	**WT allele**	**Mutant allele**	**RO**	**AO**	**Mutation type**	**Cadenza (RO/AO)**	**WT** (RO/AO)	***Ps1/ps1*** (RO/AO)	***ps1*** (RO/AO)
1D	054031	48038	1D02G123600	125830226	C	T	220	29	Silent	183/0	12/0	6/2	0/6
1D	047250	48784	1D02G137900	188459101	G	A	87	13	Missense	54/0	19/0	2/4	0/2
1D	013266	25787	1D02G155200	217637192	G	A	48	9	Missense	31/0	8/0	4/2	0/4
1D	020062	65837	1D02G250300	342849564	C	T	299	23	Silent	254/0	12/0	4/2	0/4
1D	028837	4155	1D02G257500	349745640	C	T	73	18	Missense	54/0	2/0	2/2	0/6
1D	084480	17321	1D02G266200	362010574	G	A	103	21	Missense	88/0	4/0	4/6	0/4
2B	030230	40906	2B02G419000	147489547	G	A	25	11	Splice-acceptor	1/0	8/0	12/2	0/4
2B	000458	117421	2B02G328100	16847079	C	T	150	16	Silent	129/0	2/0	4/2	0/4
2D	068080	24365	2D02G068100	28630923	G	A	41	13	Missense	32/0	2/0	2/2	0/2
3D	052096	70083	3D02G464300	91851743	C	T	142	9	Missense	120/0	8/0	4/2	n.d.
5A	016049	74310	5A02G112300	218994472	G	A	132	34	Missense	111/0	2/0	2/2	0/2
6A	086414	29647	6A02G266600	39839513	C	T	49	9	Missense	33/0	2/0	4/2	0/2
6A	081680	25236	6A02G218300	404351866	G	A	103	17	Silent	85/0	2/0	4/2	0/2
7B	032245	84895	7B02G359400	167650156	C	T	56	15	Missense	42/0	4/0	2/2	0/2
7D	056750	61140	7D02G474700	133259098	G	A	35	6	Silent	29/0	n.d	2/2	0/2

Chr. – chromosome; RO – reference allele observations; AO – alternate allele observations

* Prefix for scaffold ID is Triticum_aestivum_Cadenza_EIv1.1_scaffold_; † Prefix for gene ID is TraesCS

Table S10: List of mutations of *CAD1290* within identified region of chromosome 1D.

Gene*	Mutation ID**	Mutation type	Ref. base	Alt. base	Linked (Y/N) †	Gene Annotation***
132200	156132709	Missense	G	A	N	*SPT20-like*
135300	176389878	Missense	C	T	N	*Initiation factor-2B*
136800	183025101	Missense	C	T	N	*SLOMO*
137000	185066347	Missense	C	T	n.d.	*Transcription factor IIA*
137900	188459101	Missense	G	A	N	*CAT1*
141700	196093648	Silent	G	A	N	*GDP-fucose protein O-fucosyltransferase*
141800	196473994	Missense	G	A	N	*Glutamine synthase*
142500	198021047	Intron	G	A	n.d.	None
144400	199835740	Intron	G	A	n.d.	*DUF1338*
144800	199928182	Intron	G	A	n.d.	*FMN/FHY*
146600	202569754	Missense	G	A	N	*Cellulose synthase*
147400	203471854	Silent	G	A	n.d.	*NPF5-like*
148500	204478111	Silent	G	A	N	*Tyrosine t-RNA ligase*
148800	204629221	Missense	G	A	N	*Anaphase-promoting complex, WD40 domain*
149000	204987820	Missense	G	A	N	*35S ribosomal protein L40*
149100	205043392	Intron	G	A	n.d.	*Non-lysosomal glucosylceramidase*
149500	205763747	Silent	G	A	N	*Papain-like cysteine peptidase*
153500	212722695	Missense	G	A	N	*Pentatricopeptide repeat protein*
154700	216133970	Missense	G	A	Y	*Cytokinin riboside 5'-monophosphate phosphoribohydrolase*
155100	217144163	Intron	G	A	N	*ARF22-like*
155200	217644011	Missense	G	A	Y	*HB-2*
156000	219726304	Intron	G	A	n.d.	*Coatomer subunit gamma*
156100	220078275	Intron	C	T	n.d.	*APP2-like*
156900	221099300	Silent	C	T	n.d.	*Serine-threonine protein kinase*
159600	225703489	Missense	G	A	N	*Class VIII myosin-like*
161400	228303464	Intron	G	A	n.d.	*E3 ubiquitin-protein ligase*
161700	228814321	Intron	G	A	n.d.	*DNA topoisomerase I, type IA*
162100	230217515	Intron	G	A	n.d.	None
162200	230669102	Intron	G	A	n.d.	*Electron transfer flavoprotein-ubiquinone oxidoreductase*
162500	232344836	Missense	G	A	Y	*U1 small nuclear ribonucleoprotein 70 kDa*
163400	233914477	Missense	G	A	N	*Aminoacyl-tRNA synthetase*
164500	235569704	Missense	G	A	N	*RNA helicase*

* The prefix for the gene ID is TraesCS1D02G

** The prefix for the mutation ID is Cadenza1290.chr1D

† Denotes whether the mutation associated with the leaf and spikelet phenotypes of *Ps1/ps1* and *ps1*. *HB-D2* is highlighted in green.

‡ The gene annotations are determined based on homology to genes in Arabidopsis and/or rice, or based on protein domain sequences.

Y – Yes; N – No; n.d. – not determined

Table S11: List of class III homoeobox-domain transcription factors in hexaploid wheat.

Gene name	Gene ID	Transcript ID*
HB-A1	*TraesCS5A02G549700*	*TraesCS5A02G549700.1*
HB-B1	*TraesCS4B02G385200*	*TraesCS4B02G385200.1*
HB-D1	*TraesCS4D02G359600*	*TraesCS4D02G359600.1*
HB-A2	*TraesCS1A02G157500*	*TraesCS1A02G157500.1*
HB-B2	*TraesCS1B02G173900*	*TraesCS1B02G173900.1*
HB-D2	*TraesCS1D02G155200*	*TraesCS1D02G155200.3*
HB-A3	*TraesCS5A02G043400*	*TraesCS5A02G043400.1*
HB-B3	*TraesCS5B02G047200*	*TraesCS5B02G047200.1*
HB-D3	*TraesCS5D02G052300*	*TraesCS5D02G052300.1*
HB-A4	*TraesCS5A02G375800*	*TraesCS5A02G375800.1*
HB-B4	*TraesCS5B02G378000*	*TraesCS5B02G378000.1*
HB-D4	*TraesCS5D02G385300*	*TraesCS5D02G385300.1*
HB-A5	*TraesCS3A02G312800*	*TraesCS3A02G312800.2*
HB-B5	*TraesCS3B02G159100*	*TraesCS3B02G159100.1*
HB-D5	*TraesCS3D02G141500*	*TraesCS3D02G141500.1*

* The transcript ID refers to the variant with the correct intron/exon structure, as determined using the RNA-seq transcriptome data, and encodes a protein with the highest sequence identity to the other two homeologs.

Table S12: Segregation ratios of BC_2F_2 generation, progeny of *ps2*.

Line	WT	Paired spikelet	Total	χ^2
4.1	9	31	40	0.71
6.5	11	28	39	0.64

Table S13: Oligonucleotide sequences for KASP assays of mutations detected in *CAD1290* exome capture analysis.

Gene ID	Mutation ID*	Allele-specific sequence [RO/AO]	Common sequence
TraesCS1D02G137900	188459101	AACAAAGAAGAGGGTTGCT[G/A]	CATGGCTCTAATATTTGCTTGTT
TraesCS1D02G155200	217644011	TGAATTGGCAACTGACCTTCATC[C/T]	TGTAAGAACTTGTTACATGTCTC
TraesCS1D02G257500	349745640	ACGGGAACTTCTGATAAA[G/A]	GTAGCAGATAGCTTAGTT
TraesCS1D02G266200	362010574	CGGGTTTGTTGATGCCGCTA[G/A]	CTGAACGCCGTTGACCTGGT
TraesCS1D02G250300	342849564	ATTTGGATTGAAGTGGATG[C/T]	TGAATGATGGTGTACCTGAT
TraesCS1D02G123600	125830226	GTTACAACTCTTGACTCACGG[G/A]	TGAATCTATCACACAAACA
TraesCS2B02G328100	16847079	CCAATCCTGACTTGCCTCG[G/A]	TGGACGGCAGAAATGGGTAGT
TraesCS2B02G419000	147489547	TTGCGTTTCTACTTGTTGCA[G/A]	GTATGCGGTCCATCTCCAAA
TraesCS2D02G068100	28630923	TGTTCCAGTGTCGCAGAAA[G/A]	AAGCTCAAGTCAGGATAGACA
TraesCS3D02G464300	91851743	TATGGATGAGTTTGTGAGG[G/A]	AGCTCCCATCCACATCCAGG
TraesCS5A02G112300	218994472	GCGATTATAGAGAGAATG[G/A]	TATCTCCCCATGCACGCTGGA
TraesCS6A02G266600	39839513	ATGGTCTCGAGGAGGAAGA[G/A]	AGGTGAACAGCGTCGACCTC
TraesCS6A02G218300	404351866	GTTCGACAAATTTCTTCGAT[C/T]	GGCACAAAGGAACCTAAGA
TraesCS7B02G359400	167650156	GGGTCGTCCACCGCTGGCA[C/T]	TGGCGCAAGTCGACCCGGAC
TraesCS7D02G474700	133259098	GACGAGCGTTCTTGTTCAC[C/T]	CATGACAGCGGTTCAGGCG

* The prefix for the mutation ID is Cadenza1290.chr1D.

Table S14: Oligonucleotide sequences for KASP assays of *CAD1290* mutations in the region of chromosome 1D.

Gene ID	Mutation ID*	Allele-specific sequence [RO/AO]	Common	Linked† (Y/N)
TraesCS1D02G132200	156132709	TCAACCACTTGTAAGCAGTGTAG[G/A]	GTTAGCAGATTTCGCAAATGC	N
TraesCS1D02G136800	183025101	CCTGTGCATACATCATACCTCA[C/T]	TGACAAATGAGATCTGCAAAGTTC	N
TraesCS1D02G137900	188459101	AACAAAGAAGAGGGTTGCT[G/A]	CATGGCTCTAATATTTGCTTGTT	N
TraesCS1D02G141700	196093648	TGCTCCGTCTCGACCTC[C/T]	AACTCCTCCGAGGACGCC	N
TraesCS1D02G141800	196473994	CAACCTGGGAGTAAACAGATG[C/T]	GATGAAAGATTCGCCAAGTGC	N
TraesCS1D02G144800	199928182	GAGTACCATTGAATATCCCA[C/T]	AGCCTTCTAGTGCTCTGTTGCT	N
TraesCS1D02G148800	204629221	GAGTACCATTGAATATCCCA[C/T]	AGCCTTCTAGTGCTCTGTTGCT	N
TraesCS1D02G149000	204987820	GCTATGGTTCCTGACATGAC[C/T]	GTGAGTGTTGCCATGTACCG	N
TraesCS1D02G154700	216133970	TGAGTTGTGCAATGCAGGAG[G/A]	TCGAGCAGCTCCTCCAGG	Y
TraesCS1D02G155200	217644011	TGAATTGGCAACTGACCTTCATC[C/T]	TGTAAGAACTTGTTACATGTCTC	Y
TraesCS1D02G159600	225703489	ATTGCTGGCGTGGATGAT[G/A]	TTGTTGTGCCCCAGGTAATG	N
TraesCS1D02G162500	232344836	TGTCCATGCCTGCTGATT[G/A]	TGTCCATGCCTGCTGATTG	N
TraesCS1D02G164500	235569704	AGATACAAAAGGTGGAGAAGAAC[C/T]	TTCATGATTTCCGCAATGGC	N

* The prefix for the mutation ID is Cadenza1290.chr1D; † Denotes whether the mutation associated with the leaf and spikelet phenotypes of *Ps1/ps1* and *ps1*. Y – Yes; N – No.

Table S15: Oligonucleotide sequences for PCR, RT-qPCR and *in situ* PCR assays.

Gene	Forward sequence	Reverse sequence	Purpose
HB-A2	CCACTCCTCCAAACCCTCTAC	CATCTTTAGAGAAACGGGAGCTAG	Confirm mutation - PCR
HB-D2	CGGATAGGATAAATATCTGATCGTT	GCACATCTTTAGAGAAACGGGAGT	Confirm mutation - PCR
HB-A2	CCACTCCTCCAAACCCTCTAC	GCAACAATACCAAACGAATCC	RT-qPCR
HB-B2	CTGCTCCCAATGCTTTTATTGT	AGCATGAGCTTGATTTCAGCGCC	RT-qPCR
HB-D2	GACCACTCCAAACCCTCTAAGG	GCAACAATACCAAACGAATCC	RT-qPCR
TraesCS6D02G145100	CATGCTCTGGGATTTATCCAT	CTGGATCATTTCCGGTGC	RT-qPCR - control
TraesCS5A02G015600	TCTAAATGTCCAGGAAGCTGTTA	CCTGTGGTGCCCAACTATT	RT-qPCR - control
VRN1	GGAAACTGAAGGCGAAGGTTGA	TGGTTCTTCCTGGATCTGATATG	RT-qPCR
AP1-2	AGCTCACCGTCACCTACACC	TTGTTTGCTTGTGCTGGAGA	RT-qPCR
AP1-3	TCTATGAGTACGCCACCGACT	CACCAATTTCCCTCACTTTCA	RT-qPCR
SEP1-3	CCTCTACCAGTTCTCCTCCTCC	CATATACTCCAGATAGTTGTT	RT-qPCR
TB-B1	GCACACAGAGACACACAAGCG	CCTCGGAAGAAGAGCAACTG	RT-qPCR
TB-D1	GCGAGTTGGTCACGAATCC	CACCCCTCCTCCAATTCAG	RT-qPCR
HB-2	GGACATGAAAACAGATGGTGC	CATCTCGTAAGGGAATTGAAAG	*In situ* PCR

Dataset S1. (separate file)

Outputs of RNA-seq analysis, including tpm values for differentially expressed genes from *Ps1/ps1* and *ps1*, and GO-term outcomes.
